# Pre-transplant T-cell clonal analysis identifies CD8^+^ donor reactive clones that contribute to kidney transplant rejection

**DOI:** 10.3389/fimmu.2025.1516772

**Published:** 2025-02-06

**Authors:** Jes M. Sanders, Barbara L. Banbury, Erika L. Schumacher, Jie He, Yuvaraj Sambandam, Paul A. Fields, Lorenzo Gallon, James M. Mathew, Joseph R. Leventhal

**Affiliations:** ^1^ Department of Surgery, Division of Organ Transplantation, Comprehensive Transplant Center, Northwestern University Feinberg School of Medicine, Chicago, IL, United States; ^2^ Adaptive Biotechnologies, Seattle, WA, United States; ^3^ Department of Medicine, Division of Nephrology, Northwestern University Feinberg School of Medicine, Chicago, IL, United States; ^4^ Simpson Querrey Institute for BioNanotechnology, Northwestern University Feinberg School of Medicine, Chicago, IL, United States; ^5^ Department of Microbiology-Immunology, Northwestern University Feinberg School of Medicine, Chicago, IL, United States

**Keywords:** kidney transplant rejection, alloreactivity, T-cell receptor sequencing, T-cell mediated rejection, mechanisms of rejection

## Abstract

**Introduction:**

Responses to allogeneic human leukocyte antigen (HLA) molecules limit the survival of transplanted organs. The changes in T-cell alloreactivity that contribute to this process, however, are not fully understood. We defined a set of donor reactive T-cell clones (DRTC) with the goal to elucidate signatures of kidney allograft rejection.

**Methods:**

DRTC were identified pretransplant using an anti-donor mixed lymphocyte reaction assay: CFSE-diluting CD4^+^ and CD8^+^ DRTC were flow-sorted, and the TCR sequences were identified using Adaptive Immunosequencing. DRTC were then tracked in post-transplant biopsies, blood, and urine samples in a cohort of kidney transplant recipients.

**Results:**

In patients with an abnormal biopsy, the majority of CD8^+^ DRTC found within the allograft were detected in the circulating pre-transplant repertoire. Circulating CD8^+^ DRTC were more abundant pre- and post-transplant in patients that received non-lymphodepletional induction and developed an abnormal biopsy when compared to stable patients. Additionally, DRTC were detected as early as two weeks post-transplant in the urine of some patients, with some of these clones subsequently identified in follow-up kidney biopsy samples.

**Discussion:**

The findings of our study add to our understanding of T-cell alloreactivity following kidney transplantation and provide evidence for the role of pre-defined alloreactive T-cells in the development of allograft rejection.

## 1 Introduction

Organ transplantation remains the gold standard treatment strategy for individuals with end-stage organ failure. Despite improved immunosuppressive regimens, rejection is a significant problem that limits long-term survival of allografts. T-cells play a crucial role in immune responses that lead to acute and chronic renal allograft rejection (1). Acute T-cell mediated rejection (TCMR) is estimated to occur in up to 10% of patients (2–5), and unsuccessful treatment inevitably leads to chronic rejection, irreversible damage, and eventual graft failure (6, 7). The risk of both acute TCMR and antibody mediated rejection (AMBR) form the basis for human leukocyte antigen (HLA) matching to identify high risk organ recipient and donor pairs (8, 9). While current crossmatching methodologies can detect the presence of pre-existing donor specific antibodies (DSA), there is no cellular based assay currently in routine use that assesses T-cell alloreactivity in the solid organ transplant setting.

To this end, we first established a methodology for alloreactive T-cell repertoire clonal analysis by using a mixed lymphocyte reaction (MLR) culture and a high-throughput sequencing technology in a group of healthy patients (10). Since that time, other groups have used similar technologies to anecdotally describe mechanisms of T-cell alloreactivity in a variety of clinical settings (11–15). Morris et al. concluded tolerance following combined kidney and bone marrow transplant (CKBMT) was, at least in part, mediated by deletion of alloreactive clones (11). The same group showed that the alloimmune repertoire is highly specific to the donor-recipient pair, and most alloreactive clones circulate at relatively low frequencies (15). More recently, Aschauer et al. described an infiltration of alloreactive clones into the allograft at rejection that was distinct from that observed in the circulation (12), concluding the two repertoires exhibit unique properties. Although these investigations have been instrumental in improving our understanding of the alloimmune response, they are limited by the relatively small sample sizes. Furthermore, the results of Aschauer et al. were impacted by the use of RNA templates as opposed to DNA, which prevented their ability to accurately assess changes in frequency of individual clones within the overall bulk repertoire. Thus, the dynamics of circulating alloreactive T-cells during kidney transplant rejection and their relationship to the intragraft repertoire remains an underexplored area of organ transplant immunology.

In this prospective study, we utilized Adaptive Immunosequencing (Adaptive Biotechnologies, Seattle, WA) to comprehensively assess T-cell alloreactivity in a cohort of 54 kidney transplant recipients with the goal to identify signatures associated with the development of rejection. Pre-transplant, donor specific MLRs were performed, proliferating (CFSE-diluted) CD8^+^ and CD4^+^ recipient T-cells were sorted, and TCRβ sequences were identified by immunosequencing. The presence and frequency of pre-identified sequences (i.e., DRTCs) was then serially monitored in prospectively collected kidney, blood, and urine samples. Collectively, the results suggest higher frequency, circulating CD8^+^ alloreactive clones contribute to allograft rejection in subjects receiving non-lymphodepletional induction therapy.

## 2 Materials and methods

### 2.1 Study design

This was a single-center, non-randomized prospective observational study performed at the Comprehensive Transplant Center at Northwestern University Feinberg School of Medicine. Kidney transplant recipients were enrolled between January 2018 and December 2019. Inclusion criteria included recipients greater than 18 years of age undergoing living or deceased donor kidney transplantation. Exclusion criteria included patients with life limiting disease not of renal etiology, active malignancy (excluding non-melanoma skin cancers), and serologic evidence of infection with HIV or hepatitis B virus (i.e. HBVs-Ag positive). Induction therapy was determined based on immunological risk, age, and history of previous immunosuppression or malignancy, and included basiliximab (Simulect), alemtuzumab (Campath), or steroids alone. Subjects requiring ABO desensitization received a combination of rituximab, IVIG, and/or plasmapheresis. Primary immunosuppression consisted of a calcineurin inhibitor, anti-metabolite, and steroids if deemed necessary. Enrolled subjects were followed for one year with sample collections obtained at designated protocol visits. Clinical laboratory values, post-transplant complications, and outcomes were assessed at 3 and 12 months post-transplant. Informed consent was obtained from all subjects. The study protocol was approved by the Northwestern Institutional Review Board (IRB number: STU00206157), and no organs/tissues were procured from prisoners. Informed consent was obtained from all subjects after the nature and possible consequences of the study were explained.

### 2.2 Sample collection and processing

#### 2.2.1 Blood

Blood samples were obtained from donors and recipients prior to transplantation, and peripheral blood mononuclear cells (PBMCs) were isolated using Ficoll-Hypaque gradient centrifugation. Donor and recipient PBMCs were then used for a pre-transplant MLR culture as described below. During the post-transplant period, recipient blood samples were serially collected at 3-months, 6-months, 12-months and any potential rejection episode(s). Recipient PBMCs were isolated as above and frozen as cell pellets at -80°C for DNA isolation and immunosequencing.

#### 2.2.2 Urine

50 to 100 milliliters of urine were collected from recipients at 2 weeks post-transplant (as a baseline post-transplant), 3-months, 6-months and 12-months post-transplant, and any possible rejection episode(s). Cell components were isolated by centrifugation at 300g and were frozen as cell pellets at -80°C for DNA isolation and immunosequencing.

#### 2.2.3 Allograft

Protocol allograft biopsies were acquired at the back-table before implantation, 3-months and 12-months post-transplant, as well as any potential rejection episode (i.e., “for-cause” biopsy). The decision to perform a “for-cause” biopsy was left up to the treating physician based upon clinical presentation and laboratory values. A second pass biopsy was also obtained, which was frozen dry at -80°C for DNA isolation and immunosequencing.

### 2.3 Definition of rejection

Patients were classified into three groups based upon biopsy findings: acute rejection, borderline changes, and stable. All pathology reports were interpreted by an attending pathologist at Northwestern Memorial Hospital. Briefly, patients were categorized as “acute rejection” if there was evidence of acute rejection on biopsy (i.e., >Banff IA and/or antibody mediated rejection), “borderline changes” if there were inflammatory changes on biopsy that did not meet criteria for “acute rejection,” and “stable” if there were no inflammatory changes evident on biopsy. Stable patients and those with borderline changes were assigned a diagnosis at both the 3-month and 12-month time points. Thus, some patients that demonstrated no changes on 3-month biopsy and later developed borderline changes were categorized as “stable” at 3 months and “borderline changes” at 12 months. For patients that rejected, samples from the first episode of rejection were used for primary analyses.

### 2.4 Pre-transplant MLR and sorting of donor reactive T-cells

**Figure 1:**
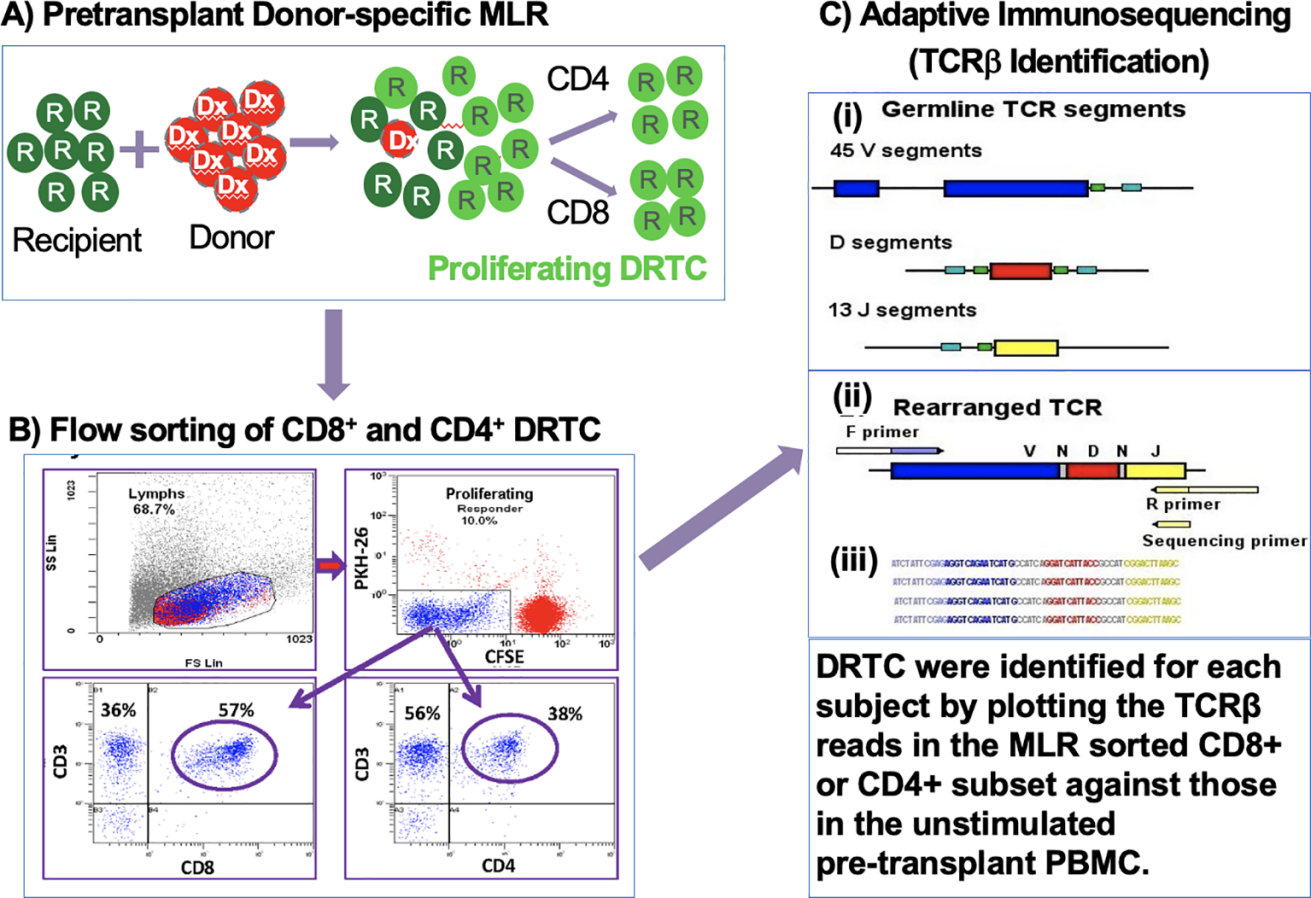
Generation, sorting, and sequencing of DRTC. **(A)** CSFE-labeled recipient PBMCs were cultured in bulk with irradiated (3000rad) PKH26-labeled donor PBMCs. **(B)** Cells were harvested, labeled with anti-CD3, anti-CD4, and anti-CD8 monoclonal antibodies and sorted for CD4^+^ and CD8^+^ subsets after gating on the CSFE-diluted cell population. **(C)** Adaptive immunosequencing was used to amplify and simultaneously sequence rearranged TCRβ sequences in a multiplex PCR.

PBMCs were isolated from recipient and donor blood samples obtained before transplantation as described above. Recipient PBMCs were labeled with CFSE, and donor cells were labeled with PKH26 and irradiated (3000 rad) as described previously (16). The labeled recipient and irradiated donor cells were then cultured in bulk in RPMI-1640 supplemented with 15% AB serum, at 37°C, 5% CO_2_, and a concentration of 1x10^6^ cells/mL (**Figure 1A**). After 7 days of culture, cells were harvested and labeled with anti-CD3, anti-CD4, and anti-CD8 monoclonal antibodies (Beckman-Coulter, Miami, FL, or Becton-Dickinson [BD], San Jose, CA). CD8^+^ and CD4^+^ donor reactive T-cells were then sorted on FACSAria (BD, San Jose, CA) by gating on the CFSE dim population (i.e., proliferating responder cells), after gating out both CFSE high cells (i.e., recipient non-proliferating cells) and residual PKH26^+^ donor stimulator cells. Phenotypic subset analysis of the flow sorting panel and CD8^+^ and CD4^+^ donor reactive T-cells are shown in **Figure 1B**. Flow-sorted T-cells were frozen as cell pellets at -80°C for future DNA isolation and immunosequencing.

### 2.5 High-throughput TCR repertoire sequencing

Adaptive Immunosequencing is a multiplex PCR-based method that amplifies the CDR3 region of the TCRβ chain using high-throughput sequencing technology with a PCR amplification bias-control process, which ensures a quantitative read-out of the immune repertoire. Since this technology utilizes genomic DNA, it can identify the frequency of common and rare CDR3 sequences, and these observed frequencies are highly representative of the frequencies in the larger population of T-cells (10).

Since this was a discovery phase project, all samples totaling 768 (including pre-transplant MLRs), were sent for immunosequencing in two batches. This was to eliminate any potential inter-assay variability, despite our initial observation that the predominant clones detected were similar between assays and over time in any individual (10). Briefly, genomic DNA was extracted from samples using the QIAamp DNA blood Mini kit (Qiagen) and amplified in a bias-controlled multiplex PCR using forward and reverse primers specific to CDR3 Vβ and Jβ region (17). The CDR3 region of rearranged TCRβ, which was defined based on IMGT/Junction Analysis (18), was then sequenced (17) (**Figure 1C** **).** Raw sequence data were preprocessed to remove errors in the primary sequence of each read and to compress the data (19). Sequences were then collapsed and filtered in order to identify and quantify the absolute abundance of each unique TCRβ CDR3 region for further analysis as previously described (17, 20). Pairwise comparisons of repertoire-wide clonal frequencies were performed as part of a quality control process to ensure no material transfer or sample swapping occurred during sample processing.

### 2.6 Serial monitoring of donor reactive T-cell clones

**Figure 2:**
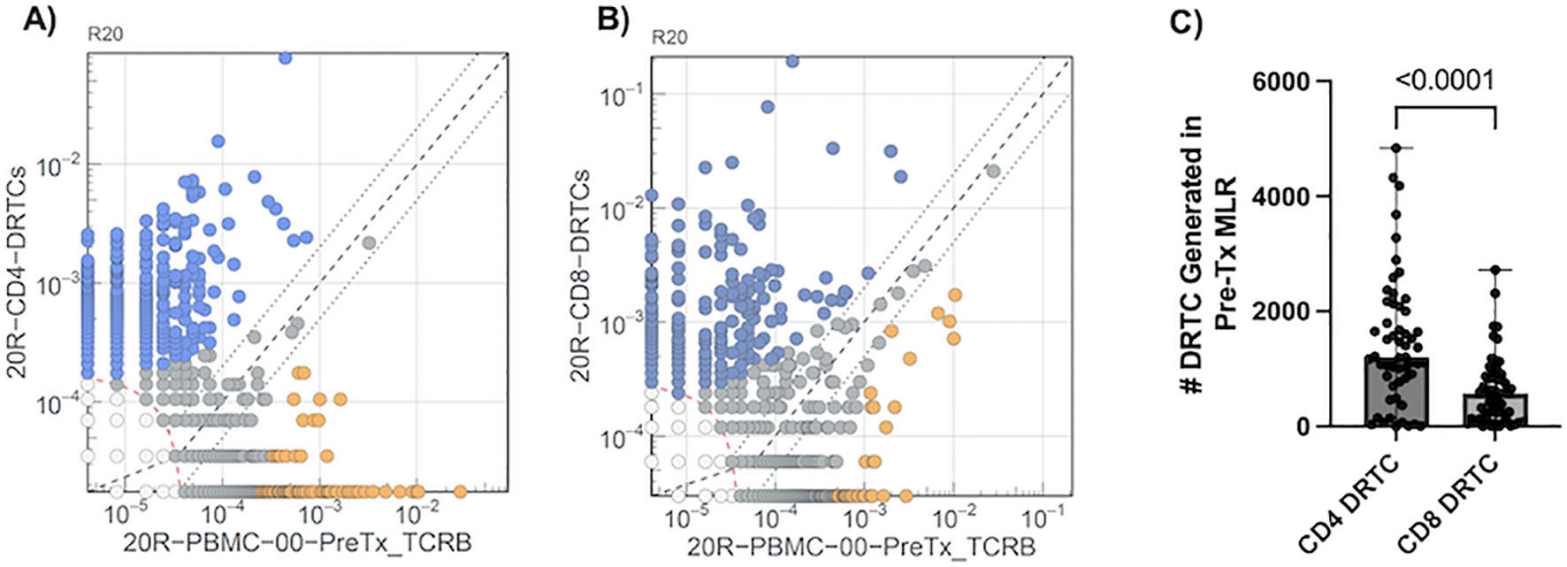
Defining DRTC Using Differential Abundance. DRTC were defined by comparing clone frequencies in the MLR sorted **(A)** CD4^+^ and **(B)** CD8^+^ samples against the unstimulated, pre-transplant sample. A representative experiment to define DRTC is depicted. TCRβ sequences detected in the MLR CD4^+^ or CD8^+^ sorted populations are plotted (Y-axis) against TCRβ sequences identified in the unstimulated pre-transplant PBMC sample (X-axis). DRTC were defined based upon criteria described in Materials and Methods. Blue circles denote DRTC. Grey circles are clones that did not meet established criteria. Orange circles are clones that are significantly more abundant in the unstimulated, pre-transplant PBMC but are likely not alloreactive as they were less frequent in the MLR sorted population compared to the unstimulated, pre-transplant PBMC sample. **(C)** Scatterplot comparing the number of CD4^+^ and CD8^+^ DRTC generated in the pre-transplant MLR in a paired fashion (N=54; Wilcoxon Signed Rank Test).

DRTC were identified utilizing differential abundance analyses generated for each subject as described previously (21) (**Figure 2A**). In brief, each rearrangement with a combined total count of at least 5 is treated as a fixed number of “trials” (Bernoulli experiments) in a two-sided binomial test. The probability, p, of the observed template counts in each sample is calculated under the null hypothesis that these templates are evenly distributed between the two samples, relative to their respective repertoire sizes (i.e., the total productive template count of each sample). Rearrangements that are more unequally distributed relative to this expected proportion will result lower probabilities. A rearrangement is considered differentially abundant if it satisfies two criteria: 1) p < 0.01 after applying the Benjamini-Hochberg adjustment procedure to control false discovery rate (FDR) (22); and 2) the rearrangement has a frequency at least 2-fold higher in one sample than in the other. In the context of DRTC, the proliferated CD8^+^ or CD4^+^ sorted cell populations were compared against the unstimulated, pre-transplant PBMC sample. DRTC were then defined as clones that were significantly, and at least 2-fold, more abundant in the MLR sort relative to the unstimulated pre-transplant sample in order to identify those that may be most biologically relevant (i.e., expand more readily post-transplant). As shown in **Figure 2A**, DRTC meeting both significance and 2-fold increase criteria for subject 20R are depicted as blue circles. DRTC metrics used for analysis included the absolute number of DRTC, the frequency of these clones within the entire T cell repertoire (i.e., depth), and proportion of all unique TCR clones detected that were DRTC (i.e., breadth). The Morisita Index (MI) and Jaccard Index (JI) were used to compare the similarities of the bulk repertoire between two, independent samples. Both indices are scored 0-1, with 0 being completely divergent repertoires and 1 being identical.

### 2.7 Statistical analyses

Demographic and clinical covariates were compared between study groups using unpaired Student T-tests for continuous and Fisher’s Exact Test for categorical variables. Mann-Whitney U-Test and Kruskal-Wallis were used for nonparametric independent, experimental samples, including comparisons of DRTC in patient samples. Wilcoxon signed rank tests were used to compare paired DRTC measurements over time. For all experimental DRTC metrics, median values are reported with associated ranges. All statistical analyses were performed in Prism (GraphPad) version 10.1.1., and p values of <0.05 were considered statistically significant.

## 3 Results

### 3.1 Generation of DRTC in a cohort of kidney transplant recipients

Of the 80 subjects enrolled into the study, 54 completed the required study protocol visits and/or provided ample samples for longitudinal tracking of donor reactive T-cell clones (DRTC). Basic cohort demographic and clinical characteristics are shown in **Table 1**, with individual subject data provided in **Supplementary Table S1**. There was variability in terms of recipient age, sex, race, etiology of disease, and immunological risk based on: degree of HLA mismatch, presence of pre-operative donor-specific antibodies (DSA), previous transplantation, and level of sensitization. Approximately 67% of subjects received Campath for primary induction therapy. These patients were younger and had lower body mass index (BMI), but otherwise did not significantly differ when compared to those that received non-lymphodepleting induction therapy (i.e., Simulect or solumedrol) (**Supplementary Table S2**). As described in Materials and Methods, differential abundance analyses were performed for each patient to select TCR sequences meeting criteria to be considered a DRTC (**Figures 2A, B**). A median of 1,210 CD4^+^ and 577 CD8^+^ DRTC were identified in the pre-transplant MLR (**Figure 2C**). At the subject level, more CD4^+^ DRTC were generated than CD8^+^ DRTC.

**Table 1:** Demographic and clinical characteristics of enrolled subjects.

	KTx Recipient (N=54)
Age (years)	47.6 ± 13.9
White; N (%)	40 (74.1%)
Other; N (%)	14 (25.9%)
Hispanic; N (%)	10 (18.5%)
Male; N (%)	35 (64.8%)
Body-Mass Index (kg/m2)	29.1 ± 5.7
Prior Transplant; N (%)	6 (11.1%)
Pre-operative Dialysis; N (%)	29 (53.7%)
Deceased Donor; N (%)	6 (11.1%)
Donor Age (years)	42.6 ± 13.8
Male Donor; N (%)	31 (57.4%)
Pre-operative DSA; N (%)	7 (13.0%)
ABO Incompatible; N (%)	1 (1.9%)
Campath; N (%)	34 (63.0%)
Simulect; N (%)	18 (33.3%)
Solumedrol; N (%)	2 (3.7%)
Rituxan; N (%)	8 (14.8%)
TPE/IVIG; N (%)	2 (3.7%)
Documented DGF; N (%)	6 (11.1%)
Dialysis at 12 months; N (%)	0 (0%)
Graft failure; N (%)	0 (0%)
Pathological Findings; N (%)			
Acute Rejection	9 (16.7%)
Borderline Changes (3 mo)	14 (25.9%)
Borderline Changes (12 mo)	17 (31.5%)
No rejection/Stable	14 (25.9%)

As previously observed by us, and others, there were DRTC newly detected in the MLR stimulated samples when compared to the unstimulated pre-transplant PBMC (10, 15). Thus, we assessed the absolute number and frequency of DRTC in the unstimulated, pre-transplant PBMC samples to acquire a pre-transplant baseline for each subject. Interestingly, the number of CD4^+^ DRTC present in the pre-transplant PBMC was positively correlated with their frequency, but this was not seen with CD8^+^ DRTC (**Supplementary Figures S1A, B**). Some subjects had very few CD8^+^ DRTC identified in the pre-transplant sample, but these DRTC were present at disproportionately high frequencies (**Supplementary Figure S1B**), suggesting the presence of low numbers and high frequency, cross-reactive memory CD8 T cells in some patients (23).

### 3.2 Circulating DRTC are differentially impacted by induction therapy

In order to assess the impact of induction therapy on circulating DRTC, patients were stratified into lymphodepleting (i.e., Campath) and non-lymphodepleting (i.e., Simulect and Solumedrol) groups, after which DRTC were tracked in the pre-transplant to 3-month post-transplant PBMC samples. Bulk T-cell repertoire metrics are shown in **Supplementary Figure S2**. Overall T-cell Fraction and the number of unique clonotypes significantly decreased in the Campath group (**Supplementary Figures S2A, C**), whereas in the Non-Campath group only T-cell Fraction significantly decreased (**Supplementary Figures S2B, D**). The Morisita Index (MI) and Jaccard Index (JI) were utilized to evaluate the similarity of the bulk repertoire between samples. The MI considers both the number of shared clones and their frequency, whereas the JI takes into account only the number of shared clones irrespective of their abundance. We found both MI and JI were significantly greater in subjects that did not receive Campath when compared to those receiving Campath (**Supplementary Figures S2E, F**).

**Figure 3:**
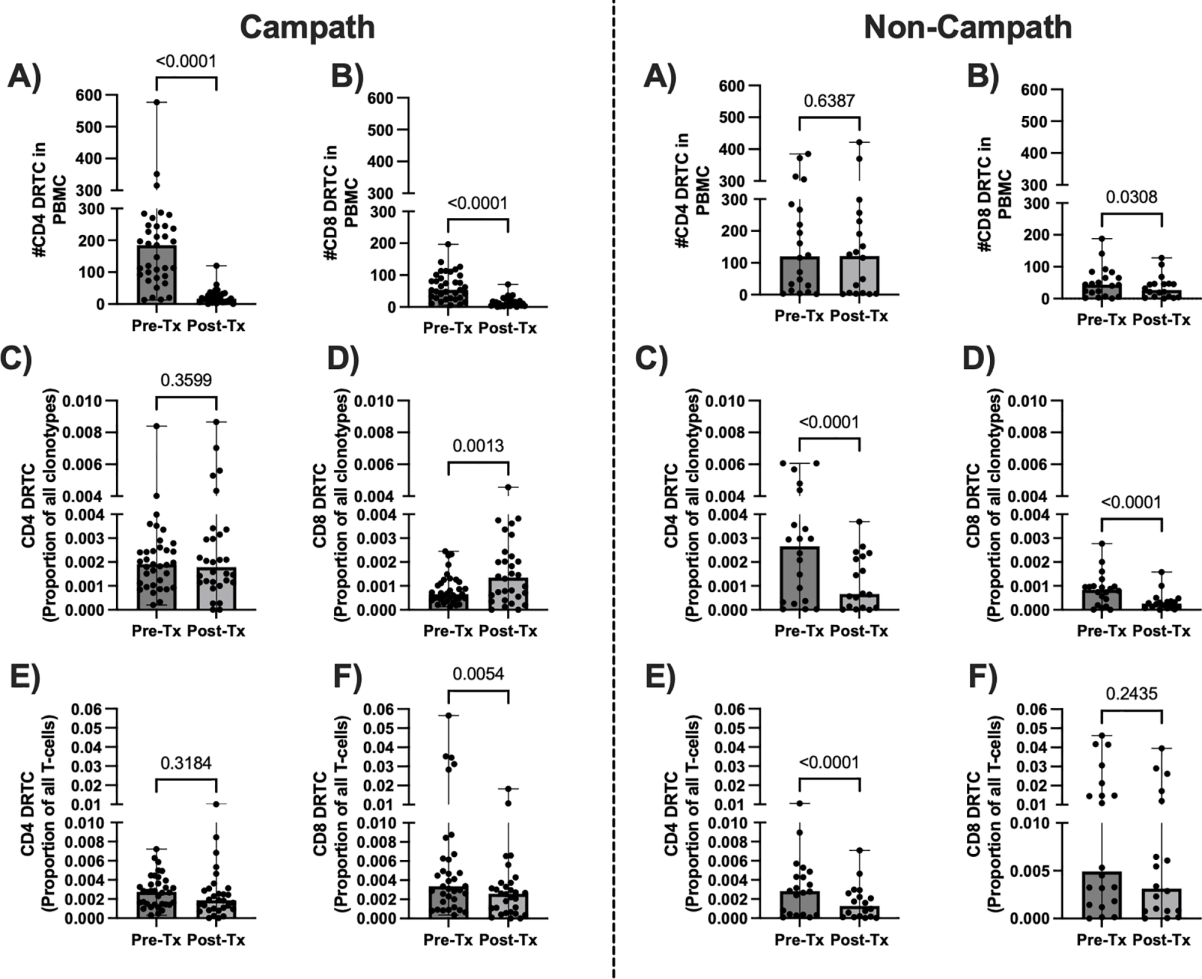
Dynamics of the circulating alloreactive repertoire from pre-transplant to 3 months post-transplant. The absolute number of DRTC, the proportion of all clonotypes (i.e., breadth), and proportion of all T-cells (i.e., frequency/depth) was evaluated in the pre-transplant (Pre-Tx) and ~3-month posttransplant (Post-Tx) PBMC samples to assess the impact of induction therapy on DRTC. Left Panel) Scatterplots comparing **(A, B)** the absolute number, **(C, D)** the proportion of all clonotypes, and the **(E, F)** the frequency of CD4^+^ and CD8^+^ DRTC in subjects that received Campath (Campath Pre-Tx N=34, Post-Tx N=30; Wilcoxon Signed Rank Test). Right Panel) Scatterplots comparing **(A, B)** the absolute number, **(C, D)** the proportion of all clonotypes, and the **(E, F)** the frequency of CD4^+^ and CD8+ DRTC in subjects that did not receive Campath (Non-Campath Pre-Tx N=20, Post-Tx N=18; Wilcoxon Signed Rank Test).

More specific to our pre-defined alloreactive repertoire, the absolute number and frequency of CD8^+^ DRTC and the absolute number of CD4^+^ DRTC decreased in patients receiving Campath (**Figure 3**, left panel). Interestingly though, in spite of the decrease in absolute number of DRTC, the proportion of unique clonotypes identified as CD8^+^ DRTC increased while CD4^+^ DRTC did not significantly change (**Figure 3**, left panel). In the Non-Campath group, the absolute number of CD8^+^ DRTC and frequency of CD4^+^ DRTC decreased, while the number of CD4^+^ DRTC and frequency of CD8^+^ DRTC was not significantly different (**Figure 3**, right panel). Both CD4^+^ and CD8^+^ DRTC constituted a smaller proportion of all unique clonotypes in the post-transplant samples compared to pre-transplant in subjects that underwent non-lymphodepletional induction (**Figure 3**, right panel).

### 3.3 Increased presence of pre-transplant circulating CD8^+^ DRTC is associated with acute rejection in subjects receiving non-lymphodepletional induction

**Figure 4:**
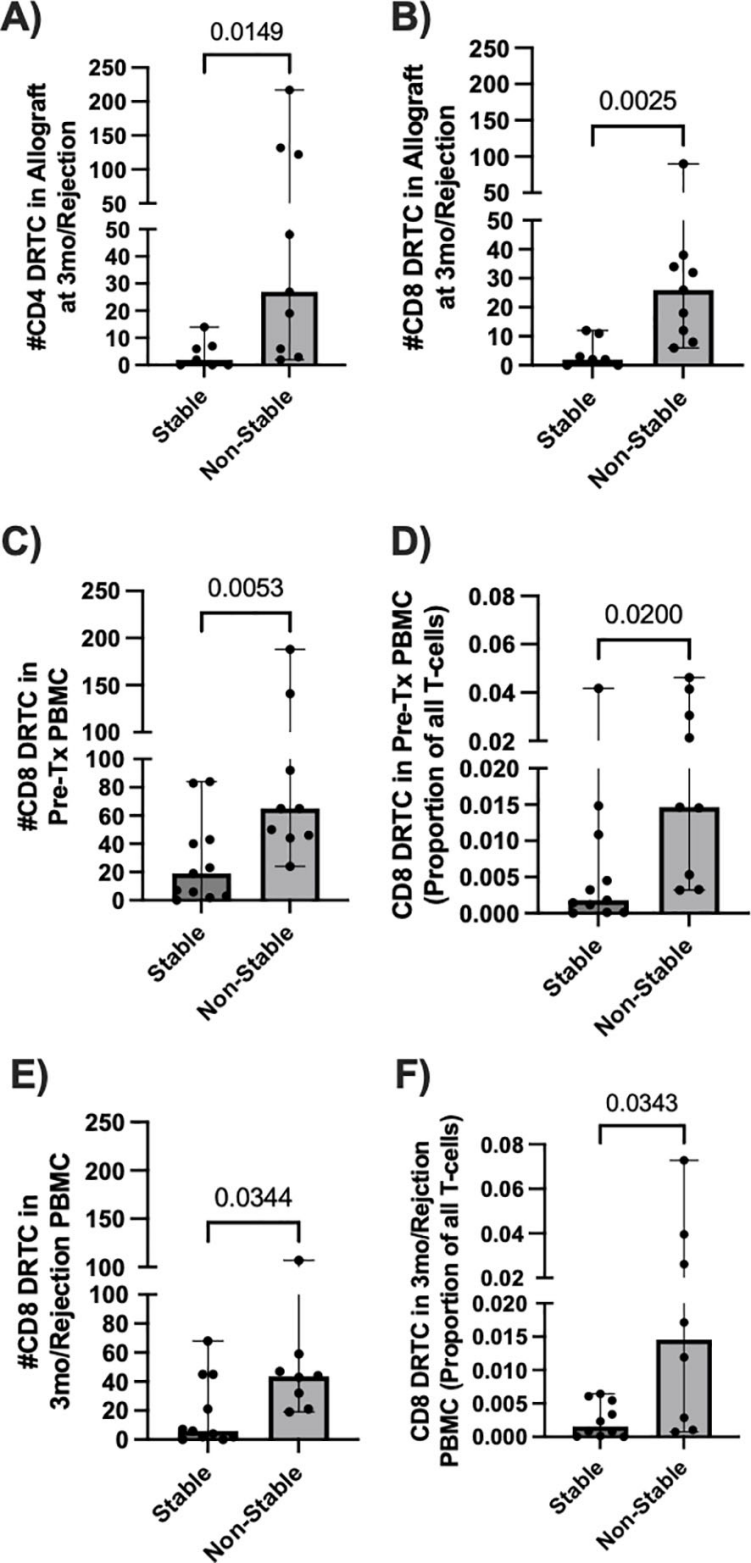
Allograft and circulating DRTC metrics associated with development of an abnormal biopsy in subjects that received non-lymphodepletional induction. DRTC were detected in pre-transplant PBMC, post-transplant allografts, and biopsy-paired post-transplant PBMC samples in order to identify T-cell changes in subjects that developed rejection/borderline rejection. **(A, B)** Scatterplots comparing the number of graft-infiltrating CD4^+^ and CD8^+^ DRTC in subjects that developed an abnormal biopsy versus those that had a normal biopsy at 3 months post-transplant (Stable N=7, Non-Stable N=9; Mann Whitney U test). Scatterplots comparing the **(C)** absolute number and **(D)** frequency of CD8^+^ DRTC in the unstimulated, pre-transplant PBMC between subjects that would subsequently develop an abnormal biopsy and those that remained stable at 3-months post-transplant (Stable N=11, Non-Stable N=9; Mann Whitney U test). **(E, F)** Scatterplots comparing the number and absolute frequency of CD8^+^ DRTC in the biopsy-paired, post-transplant PBMC sample (Stable N=10, Non-Stable N=8; Mann Whitney U test).

Due to the relative stability of the repertoire from pre- to post-transplant in the Non-Campath group (**Supplementary Figure S2**), we looked for potential signatures of rejection in this group. Additionally, due to the substantial lymphodepletion observed in subjects that received Campath induction, all remaining analyses were performed only on subjects that did not receive Campath. Given acute TCMR is associated with infiltration of alloreactive T cells into the allograft, we first examined DRTC in biopsy samples to determine the ability of our methodology to identify rejection. When compared to subjects that had a normal biopsy at 3 months post-transplant, those with rejection/borderline rejection (non-stable) had an increased number of both CD4^+^ and CD8^+^ DRTC present within the allograft (**Figures 4A, B**). In non-stable patients, CD4^+^ DRTC made up 0.9% of all clonotypes and represented 0.7% of all T cells, whereas CD8^+^ DRTC made up 0.5% of all clonotypes and represented 1.6% of all T cells. In stable subjects, CD4^+^ DRTC made up 0.6% of all clonotypes and represented 0.5% of all T cells, and CD8^+^ DRTC made up 0.3% of all clonotypes and represented 0.6% of all T cells.

Next, we investigated changes in circulating DRTC that were correlated with rejection and how they compared with graft infiltrating DRTC. In the unstimulated, pre-transplant PBMC samples, there was both an increased number and frequency of CD8^+^ DRTC in patients that subsequently developed an abnormal biopsy (**Figures 4C, D**). An increased number and frequency of CD8^+^ DRTC was also seen in the paired PBMC sample obtained at rejection (**Figures 4E, F**). However, there were no statistically significant differences between stable and non-stable patients when evaluating circulating CD4^+^ DRTC in the pre- or post-transplant periods (**Supplementary Figure S3**). We hypothesize this may be due to deletion of clones and/or an increase in the number of regulatory CD4^+^ T cells in the stable group. Among those with rejection, patients that had greater numbers of both CD4^+^ and CD8^+^ peripheral blood DRTC in the pre-transplant period tended to have greater numbers of DRTC in the post-transplant period (**Supplementary Figure S4**). This was in keeping with the data shown in **Supplementary Figure S2**. There were no clinical parameters that were associated with the increase in DRTC among those patients with rejection. However, the two patients with Banff 1A rejection at 3 months (Subject 62R, 30R) had the two highest frequencies of peripheral blood CD8^+^ DRTC in both the pre and post-transplant PBMC samples. Additionally, subject 62R had the greatest frequency of CD8^+^ DRTC in the pre-transplant peripheral blood and developed acute rejection at 2 weeks post-transplant.

**Figure 5:**
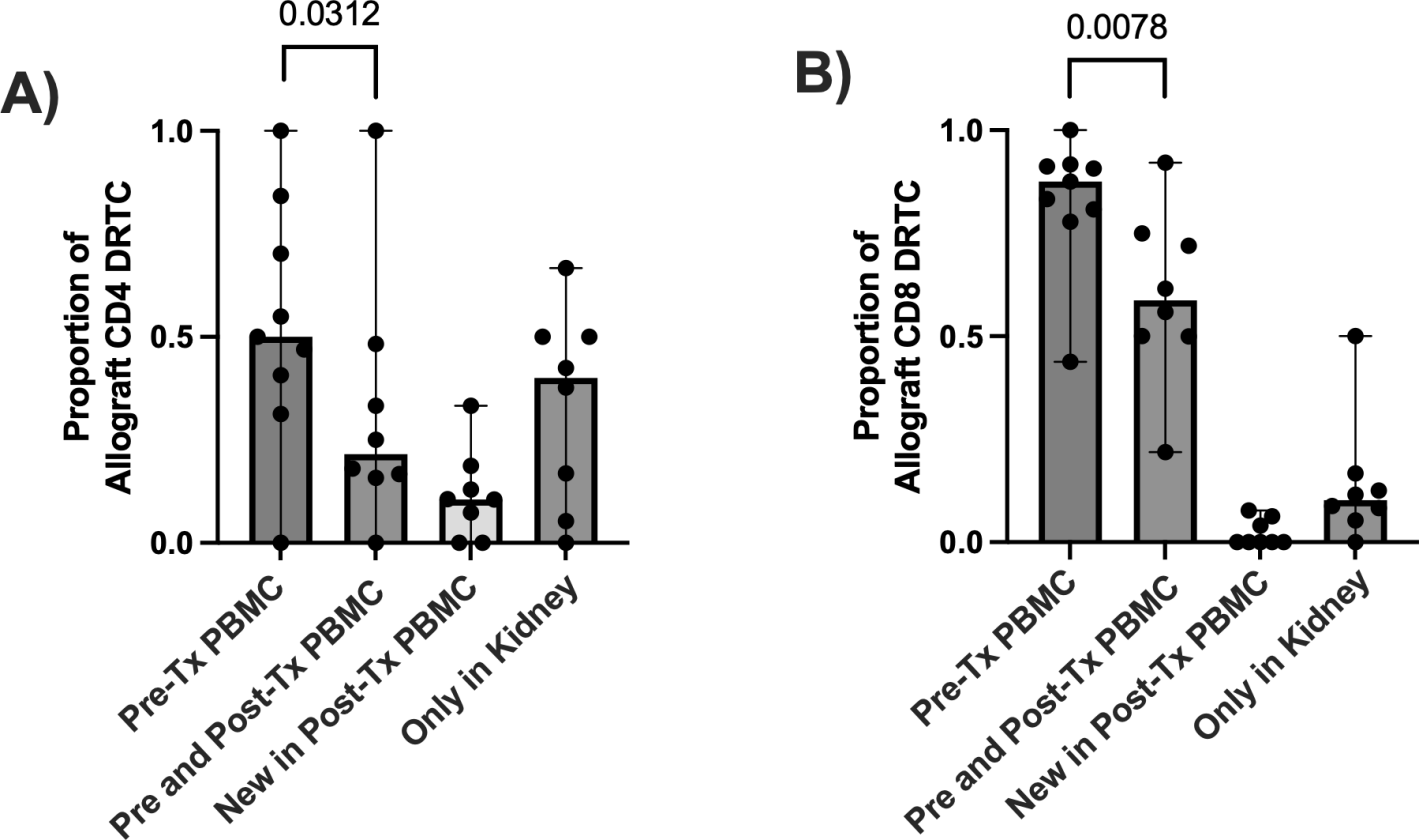
Evaluating the relationship of DRTC found within the allograft to those in the circulation in subjects that received non-lymphodepletional induction and developed an abnormal biopsy. In order to determine if DRTC found within the allograft could be also detected in the circulation (i.e., PBMC sample), the proportion of allograft clones that were identified i) in the pre-transplant (Pre-Tx) PBMC ii) in both Pre-Tx and paired, post-transplant (Post-Tx) PBMC, iii) newly in the Post-Tx period and iv) only in the kidney and never in the periphery was evaluated. Scatterplots assessing this proportion for **(A)** CD4^+^ and **(B)** CD8^+^ DRTC are shown (N=9). A proportion of 1.0 would suggest all DRTC found in the allograft at rejection were also detected at a particular time point. The majority of DRTC were found in either the Pre- or Post-Tx PBMC samples. However, there was a significant decrease in the proportion of CD4^+^ and CD8^+^ allograft DRTC that were present in the Pre-Tx PBMC AND were also found in the Post-Tx sample (Pre-Tx N=9, Post-Tx N=8; Wilcoxon Signed Rank Test).

To better understand the relationship of DRTC in the peripheral blood versus in the allograft, we evaluated the proportion of allograft infiltrating DRTC against those in the unstimulated, pre-transplant and post-transplant PBMC samples. This enabled us to categorize allograft-specific DRTC into four groups: i) those identified only in the unstimulated pre-transplant PBMC sample, ii) those identified in both the pre-transplant PBMC sample and paired PBMC sample at the time of rejection, iii) those identified newly in the paired PBMC sample at rejection (i.e., not detectable pre-transplant), and iv) those identified only in the kidney (i.e., exist at such a low frequency they were not detected in either PBMC sample) (**Figures 5A, B**). When all CD8^+^ DRTC present in the rejecting allograft was considered to be 100%, 87.5% of these clones were detected in the pre-transplant PBMC, and 59% in both the pre-transplant and paired PBMC sample at the time of rejection (**Figure 5B**). We also observed approximately 1% of allograft infiltrating CD8^+^ DRTC had likely expanded in the post-transplant period and were newly detected in the post-transplant PBMC. Lastly, ~10% of allograft infiltrating CD8^+^ DRTC were identified only in the kidney and not in either PBMC sample. In the CD4^+^ subset, 50% of allograft infiltrating CD4^+^ DRTC were detected in the pre-transplant PBMC sample with a significant decrease in this proportion in the paired, post-transplant PBMC (**Figure 5A**). Approximately 10% of allograft infiltrating CD4^+^ DRTC were newly detected in the post-transplant state, and 40% of allograft infiltrating CD4^+^ DRTC were detected only in the kidney.

Individual data from patients with acute rejection who had multiple paired samples are shown in **Supplementary Figures S5**-**S7**. Of note, the DRTC identified in the allograft in subjects with acute rejection exhibited similar dynamics in the circulation, providing additional support for the ability of the pre-transplant MLR to identify the most biologically relevant clones within the alloreactive repertoire (**Supplementary Figures S5**-**S7**). In some subjects with rejection, there was a broad infiltration of DRTC into the allograft with a concomitant decrease in their circulating frequency (**Supplementary Figure S5**), whereas in others there was expansion of a small number of clones (**Supplementary Figures S6**, **S7**).

### 3.4 DRTC can be detected in urinary samples as early as two weeks post-transplant

**Figure 6:**
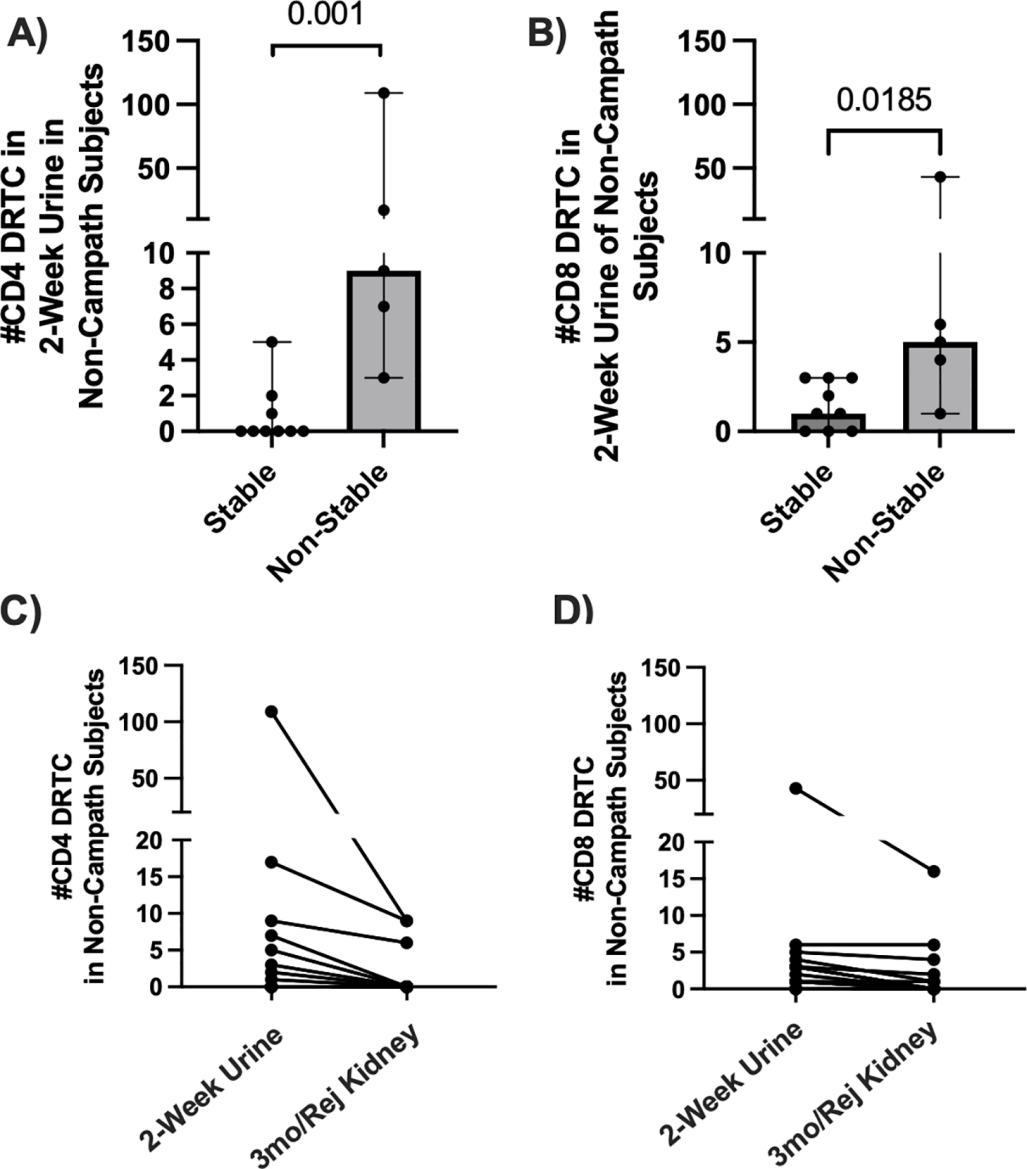
Assessment of DRTC in 2-week urine samples and tracking of these clones in post-transplant allograft biopsies. DRTC were detected in 2-week urine samples to determine if early infiltration of allografts by DRTC was associated with subsequent development of rejection/borderline rejection. **(A, B)** Scatterplots comparing the number of CD4^+^ and CD8^+^ DRTC detected in 2-week urine samples in subjects that received non-lymphodepletional induction therapy (Stable N=9, Non-Stable N=5; Mann-Whitney U Test). Subject 62R was removed from this analysis given rejection occurred at 2 weeks post-transplant. The presence of those same 2-week urine DRTC was then assessed in post-transplant allograft biopsies obtained at 3-months post-transplant or at rejection. **(C, D)** Scatterplots showing the number of CD4^+^ and CD8^+^ DRTC detected in 2-week urine samples that were also identified in follow-up kidney transplant biopsies. Overlapping CD8^+^ TCR sequences found in both the 2-week urine sample and follow-up kidney biopsy in subjects with acute rejection are shown in **Supplementary Tables S3**-**5**.

Given other groups have suggested that urinary T-cells overlap with those found within the kidney during rejection (24, 25), we evaluated the presence of DRTC in urine samples obtained serially post-transplant to determine if alloreactive clones migrated to the graft, even as early as 2 weeks post-transplant. In those subjects that did not receive Campath and subsequently developed an abnormal biopsy, 2-week CD8^+^ and CD4^+^ urinary DRTC were elevated (**Figures 6A, B**). We then tracked these 2-week urinary clones into the post-transplant period and found that some of these clones were identified in subsequent allograft biopsies performed at 3 months post-transplant or at the time of rejection/borderline rejection (**Figures 6C, D**). Overlapping CD8^+^ DRTC sequences found both in the 2-week urine and kidney biopsy samples obtained in two subjects that developed rejection (Subjects 20R, 27R) are shown in **Supplementary Tables S3**-**S5**.

## 4 Discussion

Limited studies have leveraged high-throughput TCR repertoire analyses of alloreactive T-cells in order to elucidate the dynamics of T-cell reactivity in the context of allograft rejection (11–13, 26). In this study, we utilized Adaptive Immunosequencing by Adaptive Biotechnologies to identify and characterize circulating donor reactive T-cell clones (DRTC) prior to kidney transplantation and then monitored their presence over time in the allograft, blood and urine in a cohort of kidney transplant recipients. Overall, we observed variable numbers of DRTC generated pre-transplant across individuals. The type of induction therapy had a differential impact on not only overall T-cell repertoire metrics, but also on the alloreactive repertoire. In subjects that received Campath, an increase in the number of peripheral blood unique clonotypes that were identified as CD8^+^ DRTC was increased in the post-operative state. This probably occurred as reconstitution after Campath is mediated largely by homeostatic proliferation of regulatory and memory T- lymphocytes as opposed to thymopoiesis (27, 28), resulting in a skewing of the repertoire towards a memory phenotype. Additionally, repopulation of the circulating repertoire after Campath induction is slow but CD8^+^ cells re-emerge faster than their CD4^+^ counterparts (27). Similar results have been observed in a cohort of patients receiving anti-thymocyte globulin (ATG) (26). In patients that received non-lymphodepletional induction, an amplified presence of circulating CD8^+^ DRTC in both the pre-transplant and post-transplant blood samples was associated with an abnormal biopsy. Importantly, we show the majority of CD8^+^ DRTC observed in the allograft at the time of rejection were of high enough frequency to be reliably detected in the unstimulated, pre-transplant PBMC sample. Finally, some DRTC were identified in the urine as early as 2 weeks post-transplant and were subsequently identified in the allograft at the time of rejection or 3 months post-transplant, providing evidence that alloreactive clones likely migrate to the graft in the early post-transplant period. Altogether, our results provide mechanistic insight to the development of kidney transplant rejection in subjects receiving non-lymphodepletional induction therapy and suggest monitoring of circulating CD8^+^ DRTC over time may be a surrogate for ongoing allograft inflammation.

In our pre-transplant MLR, we found that more CD4^+^ DRTC were generated than CD8^+^ DRTC. Additionally, in the unstimulated, pre-transplant PBMC sample the number of CD4^+^ DRTC identified was correlated with their frequency, but this was not true of the CD8^+^ subset. We believe these findings are probably due to highly antigenic HLA-mismatched pairs in some individuals and/or varying numbers of cross-reactive T-cells. This is in agreement with previous studies which have shown greater diversity of the alloreactive CD4^+^ compartment as opposed to the CD8^+^ (29), likely due to dominance of the CD8^+^ compartment by a small subset of highly frequent clones (15). Future studies should continue to leverage the power of deep sequencing technology to understand these nuances in specific HLA-mismatches and how they relate to rejection.

One of our main findings was that CD8^+^ DRTC were more abundant in the pre-and post-transplant peripheral blood of patients that received non-lymphodepleting induction therapy and subsequently developed an abnormal biopsy. Furthermore, we showed infiltration of these same clones into the allograft at the time of biopsy. To date, this has not been reported by other groups using similar approaches. Alackar et al. showed expansion of the TCR repertoire in the peripheral blood and allograft of patients that developed TCMR at the time of rejection, but did not specify if these clones were donor reactive (30). Similarly, in a cohort of kidney transplant recipients, TCR repertoire analysis was unable to detect a difference in the percentage of donor reactive clonotypes (i.e., breadth) within the peripheral blood of patients with rejection (12) but may have been limited by small sample size and the use of RNA templates. Alloreactive T cells are compartmentalized into both naïve and memory cell phenotypes, with memory cells arising from previous exposure to alloantigens (i.e., cross-reactivity) (31, 32). Regardless of origin, it has been shown that a high frequency of pre-existing memory T cells is associated with allograft rejection and failure (33–36) as measured by interferon-gamma (IFN-gamma) ELISPOT. This has been primarily shown in subjects that received non-lymphodepletional induction (33, 34). In our study, the number of CD8^+^ DRTC present in the pre-transplant PBMC did not necessarily correlate with their frequency. For example, in subject 62R (**Supplementary Figure S7**) there were only 46 CD8^+^ DRTC detected in the pre-transplant PBMC, but they made up a larger percentage of the bulk T-cell repertoire than any other subject that received non-lymphodepleting induction. At first rejection (~2 weeks post-transplant), the frequency of CD8^+^ DRTC increased further despite the contraction of 3 clones in the periphery (**Supplementary Figure S7**). In other subjects, there were more CD8^+^ DRTC detected in the unstimulated pre-transplant PBMC, but they made up a smaller proportion of the bulk T-cell repertoire. In such subjects (Subject 20R, **Supplementary Figure S5**), as DRTC migrated from the circulation into the allograft, there was an accompanying decline in the number and frequency of DRTC in the peripheral circulation, suggesting a broad infiltration of alloreactive clones rather than expansion of a few dominant clones. CD8^+^ DRTC in Subject 20R may also have been less antigenic and/or of a naïve phenotype, and thus, did not demonstrate the robust response expected of highly antigenic, alloreactive memory T cells (37), such as that observed in Subject 62R. Given these findings, it is necessary to consider both absolute number and frequency of clones when evaluating T-cell alloreactivity over time in transplant recipients, as mechanisms of rejection may vary across individuals. Critically, genomic DNA should be utilized for monitoring the dynamics of specific alloreactive clones as it provides more accurate estimation of clonal presence compared to RNA (12). No such differences were observed in the peripheral blood CD4^+^ DRTC when comparing stable and non-stable patients. We believe this may be due to migration of CD4^+^ DRTC into the allograft in non-stable patients, and/or deletion of clones or expansion of regulatory T cells in stable subjects. In fact, the number of detectable CD4^+^ DRTC actually increased in a number of stable patients (**Supplementary Figure S3**).

Similar to other studies, we observed an infiltration of CD4^+^ and CD8^+^ DRTC into the allograft at the time of rejection which, overall, constituted a small proportion of the intragraft repertoire (12, 24). In order to more thoroughly understand the relationship of these allograft DRTC with the circulating repertoire, we tracked graft infiltrating DRTC that could also be detected in both pre and post-transplant PBMC samples. As shown in **Figure 5**, the majority of CD8^+^ DRTC detected in the rejecting kidney biopsies were also identified in the pre-transplant PBMC samples, with a smaller proportion of allograft infiltrating CD4^+^ DRTC identified pre-transplant. We also found a significantly lower proportion of allograft infiltrating CD4^+^ and CD8^+^ DRTC that were detected in both the pre-transplant and paired PBMC samples obtained at rejection. Because of the increased presence of these clones in the pre-transplant sample, this observation is most likely a result of either i) migration of clones into the allograft with little to no recirculation, ii) selective expansion of highly alloreactive clones in the periphery with contraction of less reactive DRTC, and/or iii) deletion of a large number of clones. Equally intriguing is the fact that a substantial proportion of CD4^+^ DRTC were not detectable in either PBMC sample. We hypothesize this was due to some graft-infiltrating CD4^+^ DRTC existing at low frequencies in the periphery or residing in secondary lymph node organs, and they are only detectable once they have migrated to the allograft and undergo expansion (38). This distinction between CD8^+^ and CD4^+^ subsets could also suggest a differential role of T-cell subsets in the development of allograft rejection. Our group, and others, have discussed the potential bias of identifying high frequency, memory T cells through the use of our TCR sequencing approach (10, 15). However, the direct relationship of these circulating lymphocytes with those found in the allograft at rejection has not been thoroughly evaluated until now. We did not perform further analyses between the shared and non-shared clones in this study, but cluster and/or motif analyses of these clones may elucidate a set of epitopes important for the development of rejection and lend strength to the use of this technology as a biomarker. Importantly, due to this higher proportion of graft infiltrating CD8^+^ DRTC that exist in the unstimulated, pre-transplant PBMC, tracking changes in the CD8^+^ subset may be a more reliable marker for underlying allograft inflammation as opposed to CD4^+^ DRTC. Notably, these findings are in contrast to previous work. Aschauer et al. compared the similarity of the intragraft and circulating repertoire obtained at the time of biopsy and concluded that i) the clonal composition of the allograft was not reflected by the circulating repertoire at the time of biopsy and ii) the two repertoires exhibited distinct properties at rejection (12). However, there were limitations in that study including the lack of a comparison between the pre-transplant circulating repertoire and that of the allograft, and also that alloreactive clone analyses were only performed in two kidney biopsy samples with rejection.

Lastly, DRTC could be detected in urine samples as early as two weeks post-transplant, and in some individuals these DRTC were subsequently identified in the allograft at rejection. Although we did not perform allograft biopsies at 2 weeks post-transplant, we hypothesize the 2-week urinary clones represent a small subset of clones that migrated to the graft early in the post-transplant period, acquired a new, tissue specific phenotype, and predisposed to subsequent rejection. There is scarce prior human data, but Schenk et al. showed memory CD8^+^ T cells migrate into murine cardiac allografts as early as 24 hours of reperfusion, produce IFN-gamma, and result in downstream recruitment of primed effector T cells (39). Novel methodologies, including the use of combined single cell transcriptomics, could help further elucidate this to better understand the changes in phenotype of graft-infiltrating alloreactive clones over time (24). This would enable the development of strategies aimed at the early infiltration of CD8^+^ alloreactive clones with the goal to decrease the risk of future rejection.

There are key limitations to this study. First, we are limited by the small number and proportion of patients experiencing acute TCMR. Along with this, there are missing data points for some post-transplant samples due to the nature of prospectively collecting specimens, but we believe there were ample samples to draw meaningful conclusions. Next, given this was a discovery phase project, the study was not powered to assess the effect of changes in maintenance immunosuppressive regimens or treatment strategies on the alloreactive repertoire over time. We also did not perform in depth analyses of DRTC in subjects that received Campath given the profound lymphodepletion observed. Our cohort had a wide variation in degree of HLA mismatch, which may have an effect on DRTC generation across specific donor/recipient pairs. We cannot comment on the impact of other disease states on DRTC detection as not many patients had samples collected during episodes of CMV or BK viremia, BK nephropathy, or urinary tract infections. The mean time to rejection in our study was ~4 months, and as a result primary comparisons were performed with stable patients at 3-months. This was to control for T-cell recovery and/or expansion that occurs over time. There was variation in the number of DRTC detected across patients and sample types, further emphasizing the need for additional studies in larger cohorts. Finally, we did not report phenotypic data and can only hypothesize as to the potential role of naïve and/or memory DRTC in the development of rejection. Follow-up investigations will need to include either in-depth flow cytometric analyses or combined single cell transcriptomics to address this limitation (24). Notable strengths of the study include the prospective collection of samples (~650), comprehensive profiling of DRTC in longitudinally collected specimen types, and the use of genomic DNA allowing for exact quantification of individual clones.

In summary, high throughput TCR repertoire analysis at the DNA level was used to characterize DRTC pre-transplant, which were then prospectively monitored over time in a large cohort of kidney transplant recipients. As expected, non-lymphodepletional induction had little impact on both the bulk and pre-defined alloreactive repertoire. There was a predominance of circulating CD8^+^ alloreactive clones pre-transplant that persisted into the post-transplant state and primarily made up those CD8^+^ DRTC found within the allograft in those subjects that developed an abnormal biopsy, including acute rejection. Lastly, we show the alloreactive clones potentially home into the allograft as early as 2-weeks post-transplant, some of which are subsequently found within the allograft at later time points. The results of our study also pose an interesting question about the importance of peripheral blood versus allograft-infiltrating alloreactive clones in predicting clinical outcomes of rejection in transplant recipients. It is likely both are critical when taken in the correct clinical context. For example, identification of individuals with high frequencies of peripheral blood alloreactive clones in the pre-transplant period may suggest a greater risk of acute rejection (i.e., subject 62R in our cohort). In contrast, allograft-infiltrating clones may provide a means by which to assess treatment response when combined with traditional histopathological examination. A recent study by Shi et al. used a novel single cell technology to study alloreactivity in kidney transplant recipients experiencing rejection episodes (24). The group showed expanded clones persisted within the allograft in treatment refractory rejection, and some of these changed their phenotype and likely their function. Additional investigations should be undertaken to further define both the circulating and allograft specific alloreactive repertories as this could have substantial implications for prediction and treatment of acute rejection in transplant recipients.

## Data Availability

The datasets generated and analyzed for this study have been deposited in NCBI's Gene Expression Omnibus (GEO) and are accessible through GEO series accession number GSE288648 (https://www.ncbi.nlm.nih.gov/geo/query/acc.cgi?acc=GSE288648).

## References

[1] Mederacke YS Nienen M Jarek M Geffers R Hupa-Breier K Babel N. T cell receptor repertoires within liver allografts are different to those in the peripheral blood. J Hepatol. (2021) 74:1167–75. doi: 10.1016/j.jhep.2020.12.014 33347951

[2] Lodhi SA Lamb KE Meier-Kriesche HU. Solid organ allograft survival improvement in the United States: the long-term does not mirror the dramatic short-term success. Am J Transplant. (2011) 11:1226–35. doi: 10.1111/j.1600-6143.2011.03539.x 21564524

[3] Tanriover B Jaikaransingh V MacConmara MP Parekh JR Levea SL Ariyamuthu VK. Acute rejection rates and graft outcomes according to induction regimen among recipients of kidneys from deceased donors treated with tacrolimus and mycophenolate. Clin J Am Soc Nephrol. (2016) 11:1650–61. doi: 10.2215/CJN.13171215 PMC5012491 27364616

[4] Tamargo CL Kant S. Pathophysiology of rejection in kidney transplantation. J Clin Med. (2023) 12:4130. doi: 10.3390/jcm12124130 37373823 PMC10299312

[5] Hart A Smith J Skeans M Gustafson S Wilk A Robinson A. OPTN/SRTR 2016 annual data report: kidney. Am J Transplant. (2018) 18:18–113. doi: 10.1111/ajt.14557 29292608 PMC5772947

[6] Grimm PC McKenna R Nickerson P Russell ME Gough J Gospodarek E. Clinical rejection is distinguished from subclinical rejection by increased infiltration by a population of activated macrophages. J Am Soc Nephrol. (1999) 10:1582–9. doi: 10.1681/ASN.V1071582 10405215

[7] Nankivell BJ Borrows RJ Fung CL O’Connell PJ Allen RD Chapman JR. Natural history, risk factors, and impact of subclinical rejection in kidney transplantation. Transplantation. (2004) 78:242–9. doi: 10.1097/01.TP.0000128167.60172.CC 15280685

[8] Opelz G Dohler B. Effect of human leukocyte antigen compatibility on kidney graft survival: comparative analysis of two decades. Transplantation. (2007) 84(2):137–43. doi: 10.1097/01.tp.0000269725.74189.b9 17667803

[9] Sheldon S Poulton K. HLA typing and its influence on organ transplantation. Methods Mol Biol. (2006) 333:157–74. doi: 10.1385/1-59745-049-9:157 16790851

[10] Emerson RO Mathew JM Konieczna IM Robins HS Leventhal JR. Defining the alloreactive T cell repertoire using high-throughput sequencing of mixed lymphocyte reaction culture. PloS One. (2014) 9:e111943. doi: 10.1371/journal.pone.0111943 25365040 PMC4218856

[11] Morris H DeWolf S Robins H Sprangers B LoCascio SA Shonts BA. Tracking donor-reactive T cells: Evidence for clonal deletion in tolerant kidney transplant patients. Sci Transl Med. (2015) 7:272ra10. doi: 10.1126/scitranslmed.3010760 PMC4360892 25632034

[12] Aschauer C Jelencsics K Hu K Heinzel A Gregorich MG Vetter J. Prospective tracking of donor-reactive T-cell clones in the circulation and rejecting human kidney allografts. Front Immunol. (2021) 12:750005. doi: 10.3389/fimmu.2021.750005 34721420 PMC8552542

[13] Savage TM Shonts BA Lau S Obradovic A Robins H Shaked A. Deletion of donor-reactive T cell clones after human liver transplant. Am J Transplant. (2020) 20:538–45. doi: 10.1111/ajt.15592 PMC6984984 31509321

[14] Zuber J Shonts B Lau SP Obradovic A Fu J Yang S. Bidirectional intragraft alloreactivity drives the repopulation of human intestinal allografts and correlates with clinical outcome. Sci Immunol. (2016) 1. doi: 10.1126/sciimmunol.aah3732 PMC5323244 28239678

[15] DeWolf S Grinshpun B Savage T Lau SP Obradovic A Shonts B. Quantifying size and diversity of the human T cell alloresponse. JCI Insight. (2018) 3. doi: 10.1172/jci.insight.121256 PMC6129121 30089728

[16] Mathew JM Voss JH McEwen ST Konieczna I Chakraborty A Huang X. Generation and characterization of alloantigen-specific regulatory T cells for clinical transplant tolerance. Sci Rep. (2018) 8:1136. doi: 10.1038/s41598-018-19621-6 29348660 PMC5773708

[17] Robins HS Campregher PV Srivastava SK Wacher A Turtle CJ Kahsai O. Comprehensive assessment of T-cell receptor beta-chain diversity in alphabeta T cells. Blood. (2009) 114:4099–107. doi: 10.1182/blood-2009-04-217604 PMC2774550 19706884

[18] Yousfi Monod M Giudicelli V Chaume D Lefranc MP. IMGT/JunctionAnalysis: the first tool for the analysis of the immunoglobulin and T cell receptor complex V-J and V-D-J JUNCTIONs. Bioinformatics. (2004) 20 Suppl 1:i379–85. doi: 10.1093/bioinformatics/bth945 15262823

[19] Carlson CS Emerson RO Sherwood AM Desmarais C Chung MW Parsons JM. Using synthetic templates to design an unbiased multiplex PCR assay. Nat Commun. (2013) 4:2680. doi: 10.1038/ncomms3680 24157944

[20] Robins HS Ericson NG Guenthoer J O’Briant KC Tewari M Drescher CW. Digital genomic quantification of tumor-infiltrating lymphocytes. Sci Transl Med. (2013) 5:214ra169. doi: 10.1126/scitranslmed.3007247 PMC4026017 24307693

[21] DeWitt WS Emerson RO Lindau P Vignali M Snyder TM Desmarais C. Dynamics of the cytotoxic T cell response to a model of acute viral infection. J Virol. (2015) 89:4517–26. doi: 10.1128/JVI.03474-14 PMC4442358 25653453

[22] Benjamini Y Gavrilov Y. A simple forward selection procedure based on false discovery rate control. Ann Appl Stat. (2009) 3:179–98, 20. doi: 10.1214/08-AOAS194

[23] Ford ML Kirk AD Larsen CP. Donor-reactive T-cell stimulation history and precursor frequency: barriers to tolerance induction. Transplantation. (2009) 87:S69–74. doi: 10.1097/TP.0b013e3181a2a701 PMC2719977 19424013

[24] Shi T Burg AR Caldwell JT Roskin KM Castro-Rojas CM Chukwuma PC. Single cell transcriptomic analysis of renal allograft rejection reveals insights into intragraft TCR clonality. J Clin Invest. (2023) 133(14). doi: 10.1101/2023.02.08.524808 PMC10348771 37227784

[25] Verma A Muthukumar T Yang H Lubetzky M Cassidy MF Lee JR. Urinary cell transcriptomics and acute rejection in human kidney allografts. JCI Insight. (2020) 5. doi: 10.1172/jci.insight.131552 PMC7101135 32102984

[26] Ningoo M Cruz-Encarnacion P Khilnani C Heeger PS Fribourg M. T-cell receptor sequencing reveals selected donor-reactive CD8(+) T cell clones resist antithymocyte globulin depletion after kidney transplantation. Am J Transplant. (2023) 24:755–64. doi: 10.1016/j.ajt.2023.12.016 PMC11070313 38141722

[27] Tchao NK Turka LA. Lymphodepletion and homeostatic proliferation: implications for transplantation. Am J Transplant. (2012) 12:1079–90. doi: 10.1111/j.1600-6143.2012.04008.x 22420320

[28] Mathew JM HV J LeFever A Konieczna I Stratton C He J. A phase I clinical trial with ex vivo expanded recipient regulatory T cells in living donor kidney transplants. Sci Rep. (2018) 8:7428. doi: 10.1038/s41598-018-25574-7 29743501 PMC5943280

[29] Thome JJ Yudanin N Ohmura Y Kubota M Grinshpun B Sathaliyawala T. Spatial map of human T cell compartmentalization and maintenance over decades of life. Cell. (2014) 159:814–28. doi: 10.1016/j.cell.2014.10.026 PMC4243051 25417158

[30] Alachkar H Mutonga M Kato T Kalluri S Kakuta Y Uemura M. Quantitative characterization of T-cell repertoire and biomarkers in kidney transplant rejection. BMC Nephrol. (2016) 17:181. doi: 10.1186/s12882-016-0395-3 27871261 PMC5117555

[31] Karahan GE Claas FHJ Heidt S. Heterologous immunity of virus-specific T cells leading to alloreactivity: possible implications for solid organ transplantation. Viruses. (2021) 13. doi: 10.3390/v13122359 PMC8706157 34960628

[32] Karahan GE Claas FHJ Heidt S. Pre-existing alloreactive T and B cells and their possible relevance for pre-transplant risk estimation in kidney transplant recipients. Front Med (Lausanne). (2020) 7:340. doi: 10.3389/fmed.2020.00340 32793610 PMC7385137

[33] Hricik DE Augustine J Nickerson P Formica RN Poggio ED Rush D. Interferon gamma ELISPOT testing as a risk-stratifying biomarker for kidney transplant injury: results from the CTOT-01 multicenter study. Am J Transplant. (2015) 15:3166–73. doi: 10.1111/ajt.13401 PMC4946339 26226830

[34] Augustine JJ Poggio ED Heeger PS Hricik DE. Preferential benefit of antibody induction therapy in kidney recipients with high pretransplant frequencies of donor-reactive interferon-gamma enzyme-linked immunosorbent spots. Transplantation. (2008) 86:529–34. doi: 10.1097/TP.0b013e31818046db PMC4108983 18724221

[35] Mendoza Rojas A Verhoeven J de Kuiper R Clahsen-van Groningen MC Boer K Hesselink DA. Alloreactive T cells to assess acute rejection risk in kidney transplant recipients. Transplant Direct. (2023) 9:e1478. doi: 10.1097/TXD.0000000000001478 37096150 PMC10121441

[36] Koritzinsky EH Tsuda H Fairchild RL. Endogenous memory T cells with donor-reactivity: early post-transplant mediators of acute graft injury in unsensitized recipients. Transpl Int. (2021) 34:1360–73. doi: 10.1111/tri.13900 PMC8389524 33963616

[37] Nolz JC Starbeck-Miller GR Harty JT. Naive, effector and memory CD8 T-cell trafficking: parallels and distinctions. Immunotherapy. (2011) 3:1223–33. doi: 10.2217/imt.11.100 PMC3214994 21995573

[38] Ingulli E. Mechanism of cellular rejection in transplantation. Pediatr Nephrol. (2010) 25:61–74. doi: 10.1007/s00467-008-1020-x 21476231 PMC2778785

[39] Schenk AD Nozaki T Rabant M Valujskikh A Fairchild RL. Donor-reactive CD8 memory T cells infiltrate cardiac allografts within 24-h posttransplant in naive recipients. Am J Transplant. (2008) 8:1652–61. doi: 10.1111/j.1600-6143.2008.02302.x PMC2625311 18557725

