# Supplementary material for: Pre-transplant T-cell clonal analysis identifies CD8+ donor reactive clones that contribute to kidney transplant rejection
Source: Front Immunol. 2025 Feb 6;16:1516772. doi: 10.3389/fimmu.2025.1516772 (PMC11840674; doi:10.3389/fimmu.2025.1516772)
Supplement: Supplementary file 1 [file DataSheet1.docx]

Supplementary Material

# Supplementary Figure Legends

**Suppl. Fig. 1. Comparing absolute number and frequency of DRTC in unstimulated, pre-transplant PBMC.** DRTC were identified in the unstimulated pre-transplant PBMC sample, and the frequency of DRTC was plotted against the absolute number. The absolute number of **A)** CD4^+^ DRTC (r^2^=0.61, p=<0.0001; Spearman Correlation) but not **B)** CD8^+^ DRTC (r^2^=0.02, p=0.37; Spearman Correlation) was positively correlated with their frequency (N=54).

**Suppl. Fig. 2. Analyses of the circulating bulk T-cell repertoire pre-transplant and at 3-months post-transplant in Campath (A, C, E) and Non-Campath Subjects (B, D, F).** Bulk T-cell repertoire metrics were evaluated from pre-transplant (Pre-Tx) to 3-months post-transplant (Post-Tx) in order to determine the effect of induction therapy. **A-B)** Overall T-cell Fraction decreased from pre-transplant to 3 months post-transplant in both induction groups. **C)** The number of unique clonotypes detected in the circulating repertoire decreased in subjects that received Campath but not **D)** in subjects that did not receive Campath. In some individuals that did not receive Campath, the number of unique clonotypes increased (Campath Pre-Tx N=34, Post-Tx N=30; Non-Campath Pre-Tx N=20, Post-Tx N=18; Wilcoxon Signed Rank Test).  **E-F)** The Morisita Index (MI) and Jaccard Index (JI) was used to assess similarities between the circulating repertoire pre-and post-transplant. Both indices were higher in subjects that did not receive Campath (Campath N=30, Non-Campath N=18; Mann Whitney U Test).

**Suppl. Fig. 3. Evaluation of circulating CD4^+^** **DRTC absolute number and frequency in pre-transplant and post-transplant PBMC samples in subjects that received non-lymphodepletional induction.** DRTC were detected in pre-transplant PBMC and biopsy-paired post-transplant PBMC samples in order to identify T-cell changes in subjects that developed rejection/borderline rejection. Although the absolute number and frequency of CD4^+^ DRTC trended towards being greater in patients that had an abnormal biopsy, these differences did not reach statistical significance (Pre-Tx-Stable N=11, Non-Stable N=9; Post-Tx Stable N=10, Non-Stable N=8; Mann-Whitney U Test).

**Suppl. Fig. 4. Correlation of post-transplant PBMC DRTC metrics versus pre-transplant PBMC DRTC metrics in subjects that received non-lymphodepletional induction and developed rejection/borderline rejection.** Post-transplant PBMC metrics were plotted against the unstimulated, pre-transplant PBMC DRTC metrics for patients that developed an abnormal biopsy and received non-lymphodepletional induction. **A)** The absolute number (r^2^=0.92, p=0.0002; Spearman Correlation) and **B)** frequency (r^2^=0.98, p=<0.0001; Spearman Correlation) of CD8^+^ DRTC in the post-transplant PBMC samples was positively correlated with these metrics in the unstimulated, pre-transplant PBMC sample. Similar results were observed for **C)** the absolute number (r^2^=0.89, p=0.0005; Spearman Correlation) and **D)** frequency (r^2^=0.91, p=0.0002; Spearman Correlation) of CD4^+^ DRTC.

**Suppl. Fig. 5. Tracking of allograft DRTC in Subject 20R.**  **A-D)** Overall circulating DRTC metrics and **E-H)** dynamics of clones found in the allograft within the circulation are shown. Subject 20R received Simulect induction and was found to have borderline changes on 3-month protocol biopsy (**3**). Given there was concern for hydronephrosis, no changes were made to maintenance immunosuppression despite the changes on biopsy**.** Approximately 6.7 months post-transplant, the patient underwent for-cause biopsy due to decreasing graft function and was found to have Banff 1A rejection (**R1**). The patient was treated with a steroid burst. On follow-up biopsy ~11.5 months post-transplant, there was Banff03-IB rejection which was treated with steroids and thymoglobulin **(R2**). Of note, there was no kidney biopsy sample available at 11.5 months post-transplant for DRTC analysis. *Tracking of clones that were found in the allograft at 3 months is shown by the black dots, and clones present in the allograft at* ***R1*** *is shown by the red dots.*

**Suppl. Fig. 6. Tracking of DRTC in Subject 30R. A-D)** Overall circulating DRTC metrics and **E-H)** dynamics of clones found in the allograft within the circulation are shown Subject 30R received Simulect induction and developed Banff 1A rejection at 3 months post-transplant on 3-month protocol biopsy (**R**) and was treated with steroid burst. Following treatment, graft function remained stable, and 12-month protocol biopsy showed borderline changes. No additional changes were made to maintenance immunosuppression at 1 year post transplant. *Tracking of clones that were found in the allograft at* ***R*** *is shown by the black dots.*

**Suppl. Fig. 7. Tracking of DRTC in Subject 62R. A-D)** Overall circulating DRTC metrics and **E-H)** dynamics of clones found in the allograft within the circulation are shown Subject 62R received Simulect induction and developed Banff IA rejection approximately 2 weeks post-transplant that was treated with solumedrol and oral prednisone taper (**R1**). Follow-up biopsy approximately 1-month post-transplant showed borderline changes and given improvement no additional changes were made to maintenance immunosuppression. A for-cause biopsy approximately 9.3 months post-transplant was obtained due to rising creatinine and showed Banff IA rejection (**R2**), which was treated with solumedrol and oral prednisone taper. *Tracking of clones that were found in the allograft at* ***R1*** *is shown by the black dots***.**

# Supplementary Tables

| **Subject ID** | **Age** | **Etiology of Renal Disease** | **Race** | **Prior Transplant** | **Donor Type** | **Primary Induction** | **HLA Mismatch** | **PRA Class I** | **PRA Class II** | **Pre-Tx DSA** | **Status (3 months)** |
| --- | --- | --- | --- | --- | --- | --- | --- | --- | --- | --- | --- |
| 02R | 47 | HTN/NSAIDs | White | No | No | Campath | "5/6" | 0 | 7 | No | BRL |
| 09R | 53 | PKD | White | No | No | Campath | "5/6" | 4 | 4 | Yes | BRL |
| 26R | 48 | DM II | White | No | Yes | Campath | "5/6" | 0 | 0 | No | Reject |
| 27R | 22 | Unknown | White | No | No | Campath | "5/6" | 87 | 56 | No | Reject |
| 31R | 40 | HTN | Black | Yes | Yes | Campath | "5/6" | 94 | 75 | No | Reject |
| 37R | 57 | HTN | White | No | No | Campath | "6/6" | 7 | 4 | Yes | BRL* |
| 45R | 51 | Alports | White | Yes | No | Campath | "5/6" | 4 | 8 | No | BRL ** |
| EV 01R | 50 | HTN | White | No | No | Campath | "3/6" | 4 | 0 | No | BRL |
| EV 04R | 50 | IgA nephropathy | Asian | No | No | Campath | "3/6" | 0 | 4 | No | BRL |
| EV 07R | 19 | Alports | Decline*** | No | No | Campath | "6/6" | 0 | 0 | No | Stable |
| EV 12 | 24 | HTN | White | No | No | Campath | "5/6" | 29 | 29 | No | Reject |
| EV 17R | 45 | DM II | Decline | No | No | Campath | "5/6" | 0 | 0 | No | BRL |
| EV 25R | 42 | HTN | White | No | No | Campath | "5/6" | 0 | 0 | No | BRL |
| 01R | 38 | FSGS | White | Yes | No | Campath | "5/6" | 4 | 44 | No | Stable |
| 03R | 45 | PKD | Asian | No | NO | Campath | "4/6" | 4 | 4 | Yes | Stable |
| 08R | 55 | chronic glomerulonephritis | Decline | No | Yes | Campath | "4/6" | 14 | 0 | Yes | Stable |
| 15R | 51 | PKD | White | No | Yes | Campath | "5/6" | 92 | 0 | Yes | Stable |
| 18R | 60 | DM II | White | No | NO | Campath | "3/6" | 0 | 0 | No | Stable |
| 32R | 29 | SLE | Decline | No | No | Campath | "3/6" | 4 | 4 | No | Stable |
| 34R | 28 | FSGS | Black | NO | No | Campath | "6/6" | 0 | 0 | No | Stable |
| 44R | 45 | DM II | White | No | No | Campath | "6/6" | 0 | 0 | No | Stable |
| 46R | 41 | membranous glomerulonephritis | White | No | No | Campath | "5/6" | 0 | 0 | No | Stable |
| 53R | 31 | Agenesis | White | Yes | NO | Campath | "5/6" | 0 | 4 | No | Stable |
| 69R | 57 | RAS | White | No | No | Campath | "4/6" | 0 | 0 | No | Stable |
| EV 02R | 32 | HTN | Decline | No | No | Campath | "3/6" | 0 | 16 | No | Stable |
| EV 03R | 36 | PKD | White | No | No | Campath | "5/6" | 6 | 7 | No | Stable |
| EV 05R | 59 | IgA nephropathy | White | No | No | Campath | "3/6" | 0 | 0 | No | Stable |
| EV 08R | 54 | DM II | White | No | No | Campath | "5/6" | 7 | 0 | no | Stable |
| EV 13R | 42 | HTN | White | No | No | Campath | "6/6" | 0 | 12 | No | Stable |
| EV 14R | 55 | Thin basement membrane | White | No | No | Campath | "3/6" | 0 | 0 | No | Stable |
| EV 16R | 48 | PKD | White | No | No | Campath | "4/6" | 6 | 0 | No | Stable |
| EV 18 | 41 | Unknown | Black | No | No | Campath | "3/6" | 0 | 4 | No | Stable |
| EV 20R | 29 | IgA nephropathy | Decline | No | No | Campath | "5/6" | 0 | 0 | No | Stable |
| EV 29 | 64 | HTN, DM II | White | No | No | Campath | "4/6" | 4 | 0 | No | Stable |
| 10R | 51 | SLE | Black | No | No | Simulect | "5/6" | 0 | 9 | No | BRL |
| 13R | 46 | IgA nephropathy | White | Yes | No | Simulect | "5/6" | 0 | 0 | No | BRL |
| 20R | 68 | DM II | White | NO | Yes | Simulect | "5/6" | 0 | 0 | No | BRL |
| 23R | 35 | chronic glomerulonephritis | Decline | No | No | Simulect | "4/6" | 0 | 0 | No | BRL |
| 30R | 50 | HTN | Black | No | Yes | Simulect | "4/6" | 0 | 16 | No | Reject |
| 52R | 74 | HTN | White | No | No | Simulect | "5/6" | 4 | 0 | No | BRL |
| 57R | 47 | membranous nephropathy | White | No | NO | Simulect | "6/6" | 0 | 0 | No | BRL |
| 59R | 68 | PKD | White | No | NO | Simulect | "5/6" | 0 | 0 | No | BRL |
| 62R | 21 | chronic glomerulonephritis/SLE | White | No | No | Simulect | "3/6" | 4 | 0 | No | Reject |
| 70 R | 68 | DM II | White | No | No | Simulect | "3/6" | 0 | 0 | No | Stable |
| 16R | 69 | HTN | White | NO | No | Simulect | "6/6" | 0 | 0 | No | Stable |
| 17R | 55 | HTN | White | No | No | Solumedrol | "0/6" | 0 | 0 | No | Stable |
| 22R | 65 | DM II | Black | NO | No | Simulect | "3/6" | 0 | 0 | No | Stable |
| 28R | 64 | HTN | White | No | No | Simulect | "2/6" | 0 | 0 | No | Stable |
| 29R | 42 | DM II | White | No | No | Simulect | "0/6" | 0 | 0 | No | Stable |
| 35R | 66 | DM II | White | No | No | Simulect | "6/6" | 4 | 4 | No | Stable |
| 42R | 55 | SLE | White | No | No | Simulect | "3/6" | 4 | 0 | No | Stable |
| 48R | 23 | SLE | White | No | NO | Solumedrol | "0/6" | 0 | 0 | No | Stable |
| 49R | 58 | SLE | White | No | No | Simulect | "5/6" | 4 | 4 | Yes | Stable |
| 65R | 60 | DM II | White | Yes | NO | Simulect | "3/6" | 6 | 23 | Yes | Stable |

*Subject 37R had borderline changes on biopsy performed at 5.6 months post-transplant and had available kidney and PBMC sample

**Subject 45R had borderline changes on biopsy performed at 5.6 months post-transplant and only had a PBMC sample available.

***Subjects with “Decline” in the above table either did not report a “Race,” it was listed as “Unknown,” or they only selected Ethnicity as “Hispanic/Latino.”

**Suppl. Table 1: Individual subject data for subjects that had samples analyzed in this study.**

|  | | **Campath (N=34)** | **Non-Campath (N=20)** | **p-value** |
| --- | --- | --- | --- | --- |
| Age (years) | | 43.7 ± 11.5 | 54.3 ± 15.1 | 0.005 |
| Race; N (%) | | | | 0.53 |
|  | White | 24 (70.6%) | 16 (80%) |  |
|  | Other | 10 (29.4%) | 4 (20%) |  |
| Male; N (%) | | 24 (70.6%) | 11 55%) | 0.38 |
| Body-Mass Index (kg/m2) | | 27.6 ± 5.8 | 31.5 ± 4.6 | 0.02 |
| Prior Transplant; N (%) | | 4 (11.8%) | 2 (10%) | >0.99 |
| Pre-operative Dialysis; N (%) | | 20 58.8%) | 8 (40%) | 0.27 |
| Deceased Donor; N (%) | | 4 (11.8%) | 2 (10%) | >0.99 |
| Donor Age (years) | | 41.0 ± 13.3 | 45.4 ± 14.6 | 0.30 |
| Male Donor; N (%) | | 19 (55.9%) | 12 (60%) | >0.99 |
| Pre-operative DSA; N (%) | | 5 (14.7%) | 2 (10%) | >0.99 |
| ABO Incompatible; N (%) | | 0 (0%) | 1 (5%) | 0.37 |
| Additional Pre-operative Regimen; N (%) | | | | >0.99 |
|  | Rituxan | 5 (14.7%) | 3 (15%) |  |
|  | TPE/IVIG | 1 (2.9%) | 1 (5%) |  |

**Suppl. Table 2: Clinical characteristics of subjects stratified based upon induction therapy.**

|  | **Subject 20R Borderline Rejection Kidney at 3-Months** |  |  |
| --- | --- | --- | --- |
|  | **Nucleotide Sequence** | **Frequency in Kidney** | **Frequency in**  **2-Week Urine** |
| **CD8**  **Clone 1** | CGCACAGAGCAGGAGGACTCGGCCGTGTATCTCTGTGCCAGCAG  CTTAGCGCTAGCGGGAGCACGGGATGAGCAGTTCTTCGGGCCA | 0.000422822 | 0.001165501 |
| **CD8**  **Clone 2** | TCCACAAAGCTGGAGGACTCAGCCATGTACTTCTGTGCCAGCAG  TGAAGCGCCCCAGGATAATTATCAGCCCCAGCATTTTGGTGAT | 0.000174103 | 0.001165501 |
| **CD8**  **Clone 3** | TTGGAGCTGGAGGACTCGGCCCTGTATCTCTGTGCCACCGAAC  CTTCGGGACAGGGGGTTAACACCGGGGAGCTGTTTTTTGGAGAA | 8.70517E-05 | 0.004662005 |
| **CD8**  **Clone 4** | CTGGAGTCCGCCAGCACCAACCAGACATCTATGTACCTCTGTG  CCAGCAGCGTACAGGGGGCCCATAAGGAGCAGTACTTCGGGCCG | 1.2436E-05 | 0.001165501 |
| **CD8**  **Clone 5** | TCACCAGGCCTGGGGGACGCAGCCATGTACCTGTGTGCCACC  AGAGGGGGCCCCGACAGGGGCTATGAAAAACTGTTTTTTGGCAGT | 1.2436E-05 | 0.001165501 |
| **CD8**  **Clone 6** | NNNNTGTCGGCTGCTCCCTCCCAGACATCTGTGTACTTCTGTG  CCAGCAGTATCTCCGGGACAGGGGGCCTCTACACCTTCGGTTCG | 1.2436E-05 | 0.001165501 |

**Suppl. Table 3: TCR sequences of CD8^+^ DRTC found in 2-week urine of Subject 20R and follow-up kidney biopsy sample performed at 3 months post-transplant.**

|  | **Subject 20R Rejection Kidney at 6.7-Months** |  |  |
| --- | --- | --- | --- |
|  | **Nucleotide Sequence** | **Frequency in Kidney** | **Frequency in**  **2-Week Urine** |
| **CD8 Clone 1** | CGCACAGAGCAGGAGGACTCGGCCGTGTATCTCTGTGCCAG  CAGCTTAGCGCTAGCGGGAGCACGGGATGAGCAGTTCTTCGGGCCA | 0.000554745 | 0.001165501 |
| **CD8 Clone 2** | TCCACAAAGCTGGAGGACTCAGCCATGTACTTCTGTGCCAGC  AGTGAAGCGCCCCAGGATAATTATCAGCCCCAGCATTTTGGTGAT | 0.000301148 | 0.001165501 |
| **CD8 Clone 3** | TTGGAGCTGGAGGACTCGGCCCTGTATCTCTGTGCCACCGA  ACCTTCGGGACAGGGGGTTAACACCGGGGAGCTGTTTTTTGGAGAA | 0.000110949 | 0.004662005 |
| **CD8 Clone 4** | NNNNTGTCGGCTGCTCCCTCCCAGACATCTGTGTACTTCTGT  GCCAGCAGTATCTCCGGGACAGGGGGCCTCTACACCTTCGGTTCG | 1.58499E-05 | 0.001165501 |

**Suppl. Table 4: TCR sequences of CD8^+^ DRTC found in 2-week urine of Subject 20R and follow-up kidney biopsy sample performed at 6.7 months post-transplant.**

|  | **Subject 30R Rejection Kidney at 3-Months** |  |  |
| --- | --- | --- | --- |
|  | **Nucleotide Sequence** | **Frequency in Kidney** | **Frequency in**  **2-Week Urine** |
| **CD8 Clone 1** | CAACCTGCAAAGCTTGAGGACTCGGCCGTGTATCTCTGTGCC  AGCAGCCTCCAACGGGGCCGCACAGATACGCAGTATTTTGGCCCA | 0.003157827 | 0.018666667 |
| **CD8 Clone 2** | CACGCCCTGCAGCCAGAAGACTCAGCCCTGTATCTCTGCGCCAG  CAGCCAAGCGGACACCCCGACCTACGAGCAGTACTTCGGGCCG | 0.001675582 | 0.002666667 |
| **CD8 Clone 3** | NTGTCGGCTGCTCCCTCCCAGACATCTGTGTACTTCTGTGCCAG  CAGCCCTGTGACAGGGGTGCCCTACGAGCAGTACTTCGGGCCG | 0.001224463 | 0.002666667 |
| **CD8 Clone 4** | GTGAGCACCTTGGAGCTGGGGGACTCGGCCCTTTATCTTTGCGC  CAGCACCACCGGACAGGGGACAGATACGCAGTATTTTGGCCCA | 6.44454E-05 | 0.000888889 |

**Suppl. Table 5: TCR sequences of CD8^+^ DRTC found in 2-week urine of Subject 30R and follow-up kidney biopsy sample performed at 3 months post-transplant.**
