# Supplementary figures and images for: Pre-transplant T-cell clonal analysis identifies CD8+ donor reactive clones that contribute to kidney transplant rejection
Source: Front Immunol. 2025 Feb 6;16:1516772. doi: 10.3389/fimmu.2025.1516772 (PMC11840674; doi:10.3389/fimmu.2025.1516772)

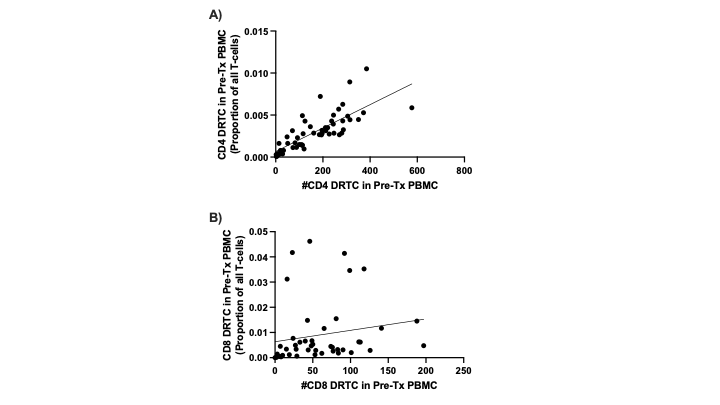

Supplement: Supplementary file 2 [file Image1.tiff]

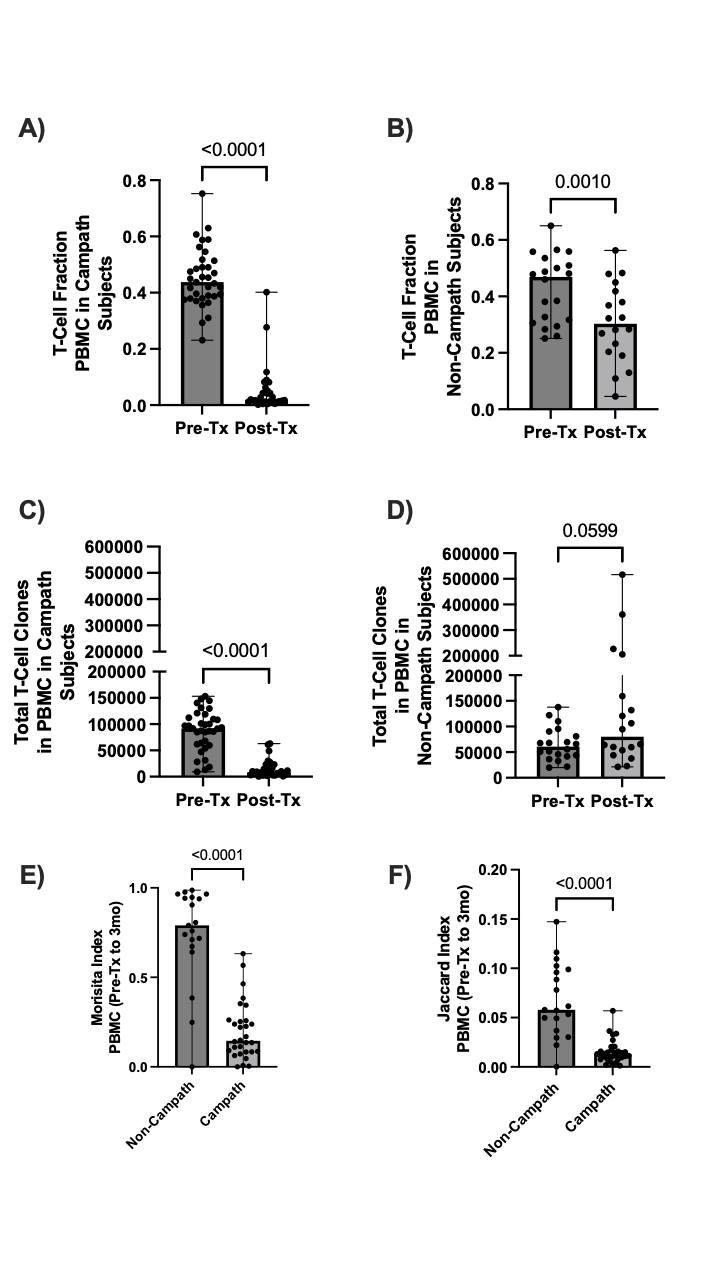

Supplement: Supplementary file 3 [file Image2.tiff]

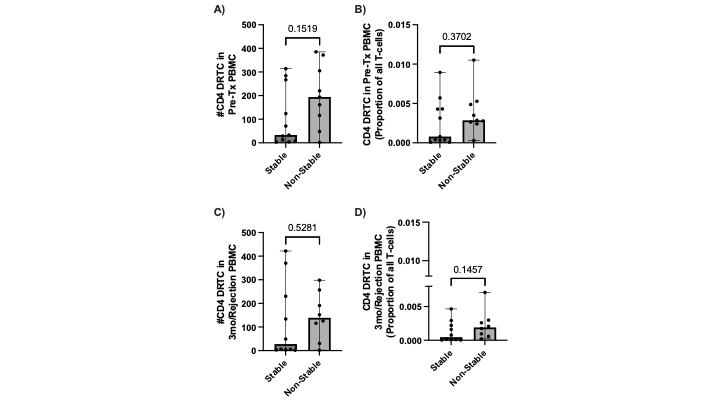

Supplement: Supplementary file 4 [file Image3.tiff]

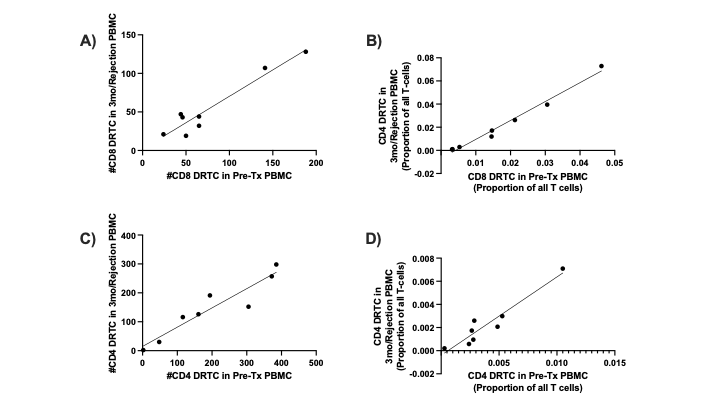

Supplement: Supplementary file 5 [file Image4.tiff]

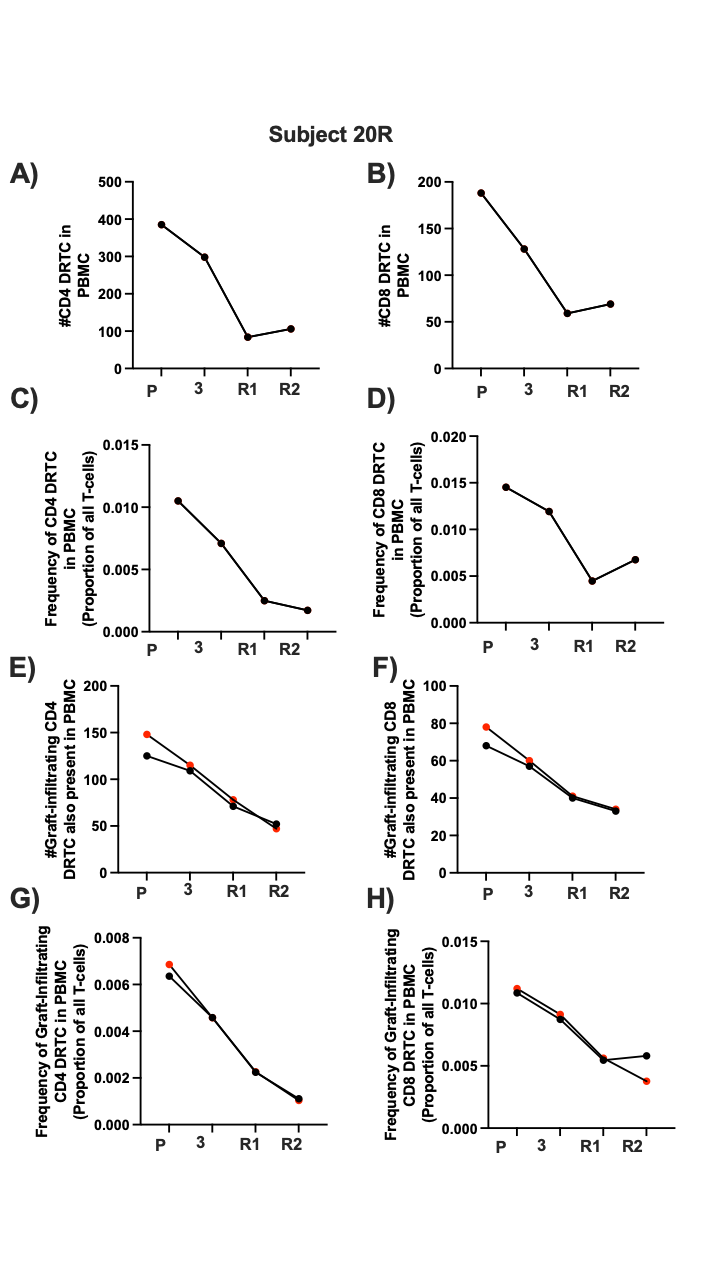

Supplement: Supplementary file 6 [file Image5.tiff]

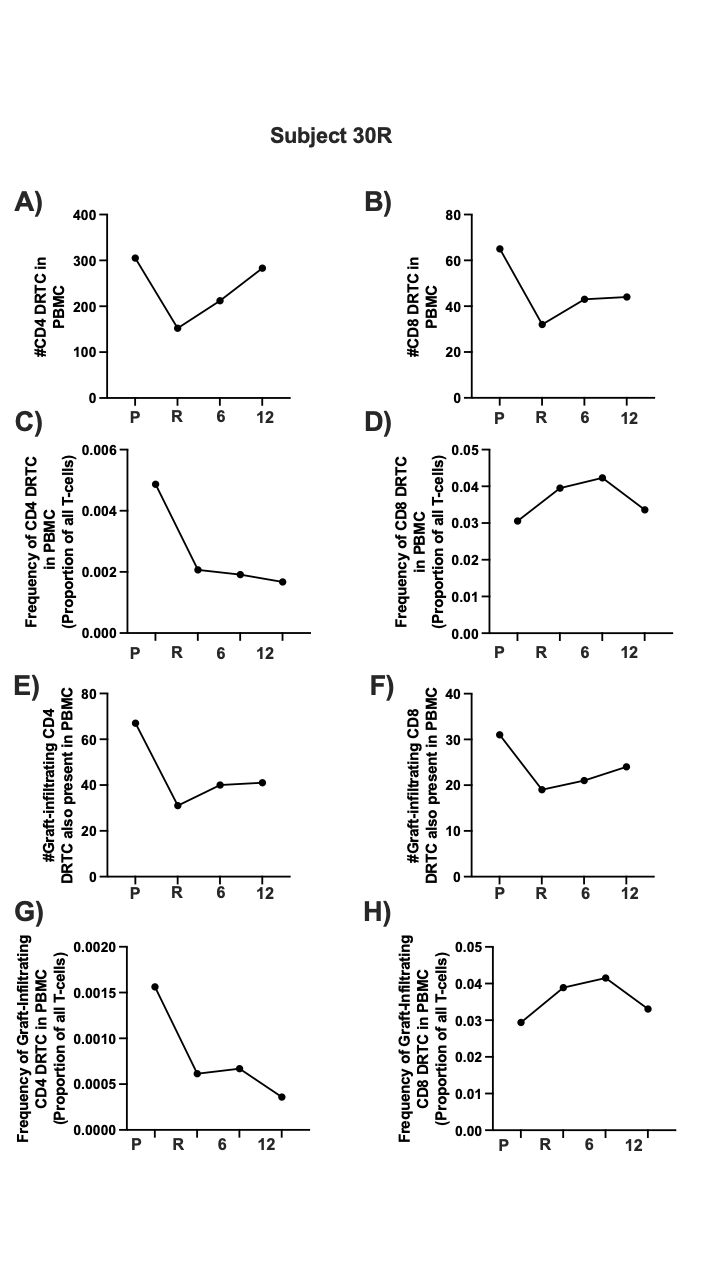

Supplement: Supplementary file 7 [file Image6.tiff]

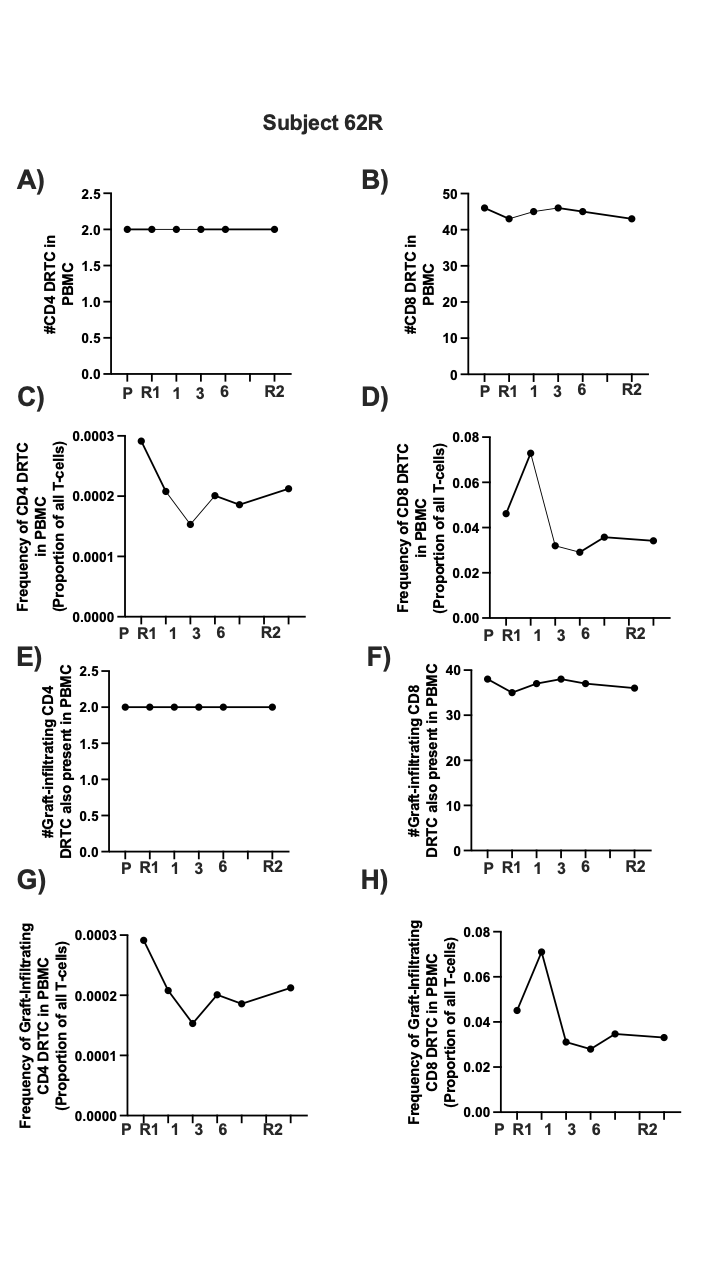

Supplement: Supplementary file 8 [file Image7.tiff]
